# HMGB3 promotes PARP inhibitor resistance through interacting with PARP1 in ovarian cancer

**DOI:** 10.1038/s41419-022-04670-7

**Published:** 2022-03-24

**Authors:** Hanlin Ma, Gonghua Qi, Fang Han, Wei Lu, Jiali Peng, Rongrong Li, Shi Yan, Cunzhong Yuan, Beihua Kong

**Affiliations:** 1 Department of Obstetrics and Gynecology, Qilu Hospital of Shandong University, Jinan, 250012 China; 2 Gynecologic Oncology Key Laboratory of Shandong Province, Qilu Hospital of Shandong University, Jinan, 250012 China; 3 School of Medicine, Cheeloo College of Medicine, Shandong University, Jinan, 250012 China; 4 Department of Ophthalmology, Qilu Hospital of Shandong University, Jinan, 250012 China

**Keywords:** Chemotherapy, Ovarian cancer, Ovarian cancer, Cancer therapeutic resistance

## Abstract

Poly (ADP-ribose) polymerase (PARP) inhibitor (PARPi) resistance remains a therapeutic challenge in ovarian cancer. High-mobility group box 3 (HMGB3) plays significant roles in the development of drug resistance of many cancers. However, the function of HMGB3 in PARPi resistance is poorly understood. In the current study, we clarified that HMGB3 was aberrantly overexpressed in high-grade serous ovarian carcinoma (HGSOC) tissues, and high HMGB3 levels indicated shorter overall survival and drug resistance in HGSOC. The overexpression of HMGB3 increased the insensitivity of ovarian cancer to PARPi, whereas HMGB3 knockdown reduced PARPi resistance. Mechanistically, PARP1 was identified as a novel interaction partner of HMGB3, which could be blocked using olaparib and was enhanced upon DNA damage conditions. We further showed that loss of HMGB3 induced PARP1 trapping at DNA lesions and inhibited the PARylation activity of PARP1, resulting in an increased DNA damage response and cell apoptosis. The PARPi-resistant role of HMGB3 was also verified in a xenograft mouse model. In conclusion, HMGB3 promoted PARPi resistance via interacting with PARP1, and the targeted inhibition of HMGB3 might overcome PARPi resistance in ovarian cancer therapy.

## Introduction

Ovarian cancer is the most common cause of cancer-related death due to gynecologic tumors worldwide [1]. Due to the difficulties in early detection, >75% of affected women are diagnosed at an advanced stage (stage III/IV), with a 5-year overall survival rate of <30% [2, 3]. In recent years, poly (ADP-ribose) polymerase (PARP) inhibitors (PARPi) have opened the door to precise ovarian cancer treatments. Three PARPi have been approved by the Food and Drug Administration (FDA) for clinical use in ovarian cancer therapy since 2014: olaparib, rucaparib, and niraparib [4]. Although PARPi have excellent anti-tumor effects, clinical studies have shown that 35% of patients are resistant to PARPi, and most sensitive patients relapse and become resistant to PARPi [5, 6]. Therefore, it is urgent to clarify the molecular mechanisms underlying PARPi resistance and construct strategies to reverse chemoresistance.

High-mobility group box 3 (HMGB3), also known as HMG2A, belongs to the HMGB family, which binds to nucleosomes in a sequence-independent manner and participates in DNA repair, replication, transcription, and recombination [7, 8]. HMGB3 is highly expressed in stem cells and cancer cells and is rarely transactivated in normal adult tissues, making it a promising therapeutic target [9, 10]. HMGB3 is reported to regulate the proper balance between hematopoietic stem cell (HSC) self-renewal and differentiation, and HMGB3 deficiency results in enhanced self-renewal capabilities in HSCs, which can induce the occurrence of leukemia [11]. Notably, HMGB3 is highly expressed and plays a vital role in the malignant progression of a variety of cancers [8]. Overexpressed HMGB3 is closely related to tumor occurrence and reduced survival in cervical cancer [12], non-small-cell lung cancer [13], breast cancer [14], bladder cancer [15], hepatocellular carcinoma [16], colorectal cancer [17], gastric cancer [18], and leukemia [19, 20]. However, the expression pattern and clinicopathological characteristics of HMGB3 in high-grade serous ovarian carcinoma (HGSOC) have not been reported.

HMGB3 also participates in the regulation of chemotherapy resistance for several tumors. Li et al. observed that HMGB3 transcriptionally activates human telomerase reverse transcriptase (hTERT) and promotes the DNA damage response, contributing to the development of radioresistance in cervical cancer [12]. HMGB3 has been revealed to be targeted by miR-27b and is associated with tamoxifen resistance in breast cancer [21]. A recent study demonstrated that the targeted depletion of HMGB3 sensitizes chemoresistant ovarian cancer cells to cisplatin through the inhibition of the ATR/CHK1/p-CHK1 DNA damage signaling pathway [22]. However, the function of HMGB3 in the development of PARPi resistance in ovarian cancer remains unclear.

In the current study, we attempted to clarify the expression and clinicopathological features of HMGB3 in patients with HGSOC. In addition, we aimed to illuminate the role played by HMGB3 in the PARPi resistance of ovarian cancer and to further elucidate the potential molecular mechanisms underlying PARPi resistance.

## Materials and methods

### Patients and samples

A total of 234 cases of HGSOC and 54 cases of fallopian tube (FT) tissues collected at Qilu Hospital of Shandong University between April 2009 and July 2015 were used to assess the expression of HMGB3. HGSOC tissues were obtained from primary ovarian cancer patients with no prior history of surgery or chemotherapy. The FT specimens were derived from patients diagnosed with benign gynecologic tumors who received hysterectomy and bilateral salpingo-oophorectomy. The clinicopathological characteristics of these patients are summarized in Table 1.

**Table 1:** Correlation between HMGB3 expression and clinicopathological characteristics.

Clinicopathological features		HMGB3 expression		
		Low expression	High expression	*p* value
Ages (years)	<56	33	45	0.7095
	≥56	70	86	
FIGO staging	I + II	24	32	0.8411
	III + IV	79	99	
CA125 (U/ml)	<600	44	54	0.9727
	≥600	51	62	
Lymph node metastasis	Positive	28	49	0.0109
	Negative	37	27	
Omentum metastasis	Positive	59	68	0.6165
	Negative	33	44	
Platinum Status	Resistance	13	32	0.0179
	Sensitive	25	22	

### Cell lines and cell culture

The UWB1.289 and SKOV3 cell lines were purchased from the American Type Culture Collection (ATCC, Manassas, VA, USA). A2780 cells were kind gift from Prof. Jianjun Wei’s Lab at Northwestern University. HEK293T was purchased from the Cell Bank of the Type Culture Collection of the Chinese Academy of Sciences (Shanghai, China). All cell lines were authenticated according to the short tandem repeat (STR) profile. UWB1.289 and A2780 were cultured in RPMI 1640 (Gibco, Grand Island, NY, USA) supplemented with 10% fetal bovine serum (FBS, Gibco). HEK293T was maintained in Dulbecco’s modified Eagle’s medium (DMEM) containing 10% FBS. SKOV3 and SKOV3/Ola cell lines were maintained in McCoy’s 5 A medium supplemented with 10% FBS. All cells were cultured in a humidified incubator at 37 °C with 5% CO_2_, and the culture media was supplemented with 1% penicillin/streptomycin (15140-122, Gibco).

### Antibodies and reagents

Antibodies against glutathione-S-transferase (GST, 10000-0-AP), poly (ADP-ribose) polymerase 1 (PARP1, 13371-1-AP), Flag (20543-1-AP), MYC-tag (16286-1-AP), GFP (66002-1-Ig), Ki-67 (27309-1-AP), histone-H3 (17168-1-AP), topoisomerase 1 (TOP1, 20705-1-AP), and caspase 3 (19677-1-AP) were purchased from Proteintech (Wuhan, China); PAR (AM80), β-actin (A5441), and methyl methanesulfonate (MMS, M4016) were obtained from Sigma–Aldrich (St. Louis, MO, USA); mouse IgG (A7028) and rabbit IgG (A7016) were purchased from Beyotime (Shanghai, China). Antibodies for γH2AX (ab22551), H2AX (ab229914), HMGB3 (ab75782), and Rad51 (ab63801) were obtained from Abcam (Cambridge, UK). Goat anti-rabbit IgG Alexa Fluor-488 (A-11008) and Goat anti-mouse IgG Alexa Fluor-594 (A-11030) for immunofluorescence staining were obtained from Invitrogen (Waltham, MA, USA). Olaparib (AZD2281) was purchased from Selleck Chemicals (Houston, TX, USA).

### Western blot

Western blot assay was conducted as previously described [23]. In brief, the tissue samples and cells were homogenized and lysed in RIPA Lysis Buffer (P0013B, Beyotime) and Western and IP Lysis Buffer (P0013, Beyotime) with 1 mM phenylmethylsulfonyl fluoride (PMSF). The cell lysate was centrifuged at 4 °C for 15 min, and the cell supernatant was collected. The concentration of extracted proteins was measured using the bicinchoninic acid (BCA) Protein Assay Kit (P0012, Beyotime). A total of 40–80 µg proteins were separated using sodium dodecyl sulfate-polyacrylamide gel electrophoresis (SDS-PAGE), followed by transfer to 0.22 µm polyvinylidene difluoride (PVDF) membranes (Merck Millipore, Burlington, MA, USA). The membranes were incubated with 5% fat-free milk for 1 h at room temperature (RT), followed by incubation with the primary antibodies (1:1000) overnight at 4 °C and appropriate horseradish peroxidase-linked secondary antibodies (1:5000) for 1 h at RT. Immunoreactive species were visualized using enhanced chemiluminescence (ECL) detection Kit (ORT2655, PerkinElmer, Waltham, MA, USA) and GE Amersham Imager 600 (GE, Chicago, IL, USA). β-actin was used as a loading control to normalize protein loading. The intensities of immunoreactive bands were analyzed using ImageJ 1.52a software from the National Institutes of Health (Bethesda, MD, USA).

### Co-immunoprecipitation (Co-IP)

The indicated plasmids were co-transfected into HEK293T cells using Lipofectamine 2000 reagent (11668-019, Invitrogen) according to the manufacturer’s instructions. In brief, the plasmids and Lipofectamine 2000 were separately incubated with Opti-MEM medium (31985070, Gibco) for 5 min at RT, after which these two reagents were mixed together, incubated at RT for 15 min, and added into HEK293T cells for 48 h. After being washed with phosphate-buffered saline (PBS), the HEK293T cells were lysed using Western and IP Lysis Buffer and centrifuged at 4 °C for 15 min. The cell supernatant (800 µg) was incubated with primary antibodies or IgG (control) overnight, followed by incubation with protein A/G agarose beads (P2012, Beyotime) for 1 h at 4 °C in a rotary shaker. The agarose beads were rinsed three times with PBS containing PMSF and resuspended with 2 × SDS loading buffer. The immunoprecipitated proteins were then subjected to western blot assay.

### Flow cytometry assay

Treated cells were washed with PBS, digested using trypsin, rinsed in PBS, and then resuspended in 1 × Binding Buffer (556547, BD Bioscience, Franklin Lakes, NJ, USA). Cells (5 × 10^5^) were incubated with fluorescein isothiocyanate (FITC)-Annexin V (5 µL) for 25 min and propidium iodide (PI, 5 µL) for 15 min in the dark at 4 °C. Flow cytometry assay was performed using a BD FACSCalibur flow cytometer, and FlowJo v10.6.2 software was used for data analysis.

### Colony formation assay

Cells were seeded onto 6-well plates at 600 cells/well and cultured in an appropriate medium containing 10% FBS. After incubation with the indicated concentrations of olaparib for 7–14 days, the colonies were fixed with methanol for 15 min, stained with 0.5% crystal violet for 15 min, and photographed. The number of colonies (>50 cells) was counted using ImageJ 1.52a software.

### MTT assay

Treated cells were plated in 96-well plates and incubated with 20 µL MTT solution (5 mg/mL) for 4 h at 37 °C. After incubation, the medium was removed, and 200 µL DMSO was added. The plate was then gently vortexed for 10 min at RT in the dark. The absorbance of the dye solution at 490 nm was measured using a microplate reader (Thermo Scientific, Waltham, MA, USA).

### Immunohistochemistry (IHC)

Human tissues and tumor xenograft tissues were fixed with formalin and embedded in paraffin. Following deparaffinization in xylene, rehydration in graded ethanol, and heat-induced antigen retrieval, 4–6-µm-thick tissue sections were incubated with primary antibodies (1:100) at 4 °C overnight, followed by incubation with the corresponding secondary antibodies, visualization using DAB (ZSGB-BIO, Beijing, China), and counterstaining with hematoxylin. The intensity of staining was scored semi-quantitatively as 0 (negative), 1 (weakly positive), 2 (moderately positive), or 3 (strongly positive). The extent of staining was based on the percentage of positive tumor cells: 1 (0–25%), 2 (26–50%), 3 (51–75%), and 4 (76–100%). The IHC grade was calculated as follows: staining intensity score × positive proportion score. The final score for each sample was the average score for two duplicates. Samples with an IHC grade ≥ 3 were considered high-expression-level samples, whereas those with an IHC grade < 3 were considered low-expression-level samples. Images were captured under a microscope (Olympus, Tokyo, Japan) using CellSens Dimension software.

### qRT-PCR

Total RNA was isolated from cells using TRIzol reagent (15596018, Invitrogen), and reverse-transcription was performed with PrimeScript RT reagent Kit (RR037A, TaKaRa, Kyoto, Japan). Real-time quantitative polymerase chain reaction (qRT-PCR) was performed using SYBR Premix Ex Taq (RR420A, TakaRa) with the 7900HT Fast Real-Time PCR System (Applied Biosystems, Waltham, MA, USA). The expression of specific genes was normalized against that of β-actin using the comparative Ct method (2^−ΔΔCt^). The primer sequences are presented in Table S1.

### Plasmid construction and lentivirus production

The PCMV-HMGB3 and PCMV-PARP1 (Flag-tag) plasmids were generated by inserting the open reading frames (ORFs) of HMGB3 and PARP1 into pLenti-C-Myc-DDK-IRES-Puro vector (PCMV, PS100069, OriGene, Rockville, MD, USA). The Myc-HMGB3 and GFP-PARP1 plasmids were generated by cloning the ORFs of HMGB3 and PAPR1 into the pMyc-C2 and pGFP-C2 vectors, respectively. pGFP-C2 and pMyc-C2 vectors were kind gifts from Prof. Zhigang Xu’s Lab at Shandong University. The accuracy of the plasmids was confirmed by DNA sequencing. The pLKO.1 HMGB3 small hairpin RNA (shRNA) vector (TRCN0000018520) and control PLKO.1 vector were purchased from Sigma-Aldrich. Lentivirus particles were produced in HEK293T cells, packaged using psPAX2 and pMD2.G. To acquire stable transfection cell lines, cells were infected with lentivirus for 24 h and then selected for 3–7 days in the corresponding culture medium containing 2 µg/mL puromycin (P8833, Sigma–Aldrich).

### Immunofluorescence

Cells seeded on glass slides were fixed with 4% paraformaldehyde for 15 min at RT, permeabilized with 0.2% Triton X-100 for 5 min at RT, and blocked with normal goat serum for 30 min at RT before being incubated with appropriate primary antibodies (1:100) overnight at 4 °C and corresponding secondary antibody (1:200) for 1 h at 37 °C. The nuclei were counterstained with DAPI for 5 min at RT. The slides were covered with antifade mounting medium and coverslips. Images were collected using an X81 microscope (Olympus).

### Tumor formation assay in nude mice

Female BALB/c nude mice (4–6 weeks) were purchased from the NBRI of Nanjing University (Nanjing, China) and raised in a pathogen-free environment with a 12-h day-night cycle. After being harvested, washed, and resuspended in PBS, 150 µL UWB1.289 cells (5 × 10^6^ cells) were subcutaneously injected into the left armpit of each mouse. Olaparib was dissolved in DMSO and diluted to 5 mg/mL with PBS before injection, and 10% DMSO in PBS was used as the vehicle control. When the tumor volumes reached ~50 mm^3^, the mice were evenly divided into four groups (PLKO.1, shHMGB3, PLKO.1 + Ola, shHMGB3 + Ola) that each received an intraperitoneal injection of olaparib (50 mg/kg, once a day). Twenty days post-injection, the mice were sacrificed, and mouse body weight and tumor volumes (length × width^2^ × 0.5) were qualified.

### GST pull-down assay

cDNA encoding HMGB3 was cloned into the pGEX-4T-1 vector to generate the GST-HMGB3 fusion vector. The GST-tagged fusion protein was amplified by isopropylthiogalactoside (IPTG)-induced expression in the *Escherichia coli* BL21 strain and purified using BeyoGold™ GST-tag Purification Resin (P2250, Beyotime), following the manufacturer’s instructions. UWB1.289 cells (1 × 10^7^) transfected with PCMV-PARP1 (flag-tag) were collected using Western and IP Lysis Buffer. After centrifugation (12,000 rpm) for 5 min at 4 °C, the cell supernatant was incubated with a GST-HMGB3 fusion protein (25 µg) or GST-tag alone in GST pull-down protein binding buffer containing GST-tag Purification Resin for 2 h at 4 °C. After washing with GST pull-down protein binding buffer two times, the supernatant was discarded, and the GST-tag Purification Resin was boiled in 2 × SDS loading buffer for 5 min. The pulled-down protein was detected using western blot.

### PARP1 enzyme activity assay

The PARP1 enzyme activity was measured using a commercial assay kit (EMD Millipore, 17-10149). In brief, GST-HMGB3 protein (50 ng) or GST protein was incubated with activated DNA (50 ng), β-NAD (0.5 mM), and recombinant PARP1 protein (100 ng) in total 20 μL of 1 × assay buffer at RT for 30 min according to the manufacturer’s protocol. After incubation with the substrate, PARP1 enzyme activity was measured by a fluorescent plate reader.

### Mass spectrometry (MS) assay

UWB1.289 (1 × 10^7^) cell protein lysates were prepared using Western and IP Lysis Buffer, followed by centrifugation at 4 °C for 15 min. The cell supernatant was immunoprecipitated (IPed) using anti-IgG or anti-HMGB3 (ab75782, Abcam) antibody. The IPed proteins were resuspended with 2 × SDS loading buffer and subjected to SDS-PAGE assay. Gels were stained with coomassie brilliant blue, and pieces were removed for MS analysis. Liquid chromatography-tandem MS (LC-MS/MS) analysis was performed on a Q Exactive mass spectrometer (Thermo Scientific) by Applied Protein Technology (Shanghai, China).

### Isolation of chromatin-bound and soluble nuclear fractions

A2780 and UWB1.289 cells transfected with PLKO.1 and HMGB3 shRNA were treated with olaparib (A2780, 4 µM; UWB1.289, 2 µM) for 4 h, followed by treatment with 4 mM MMS for 20 min. The chromatin-bound and soluble nuclear fractions of cells were separated with a Subcellular Protein Fractionation Kit for Cultured Cells (78840, Thermo Scientific), according to the manufacturer’s instructions. The protein levels of PAPR1 in chromatin-bound fractions were subjected to western blot assay.

### Development of olaparib-resistant cell lines

Olaparib-resistant SKOV3 (SKOV3/Ola) cells were induced by exposing cells to increasing concentrations of olaparib in our Lab. In brief, SKOV3 cells were cultured in McCoy’s 5 A medium containing olaparib at a start concentration of one-tenth of the IC_50_ value. Increase the concentration of olaparib every 2–3 weeks, and each increase of about 1.5 times the dose. Maintain the medication pressure for 6–10 months.

### Statistical analysis

All data were verified by performing at least three independent experiments. All experimental data are represented as the mean ± the standard error of the mean (SEM).

Statistical significance was assessed using the Student’s *t* test for comparison between the two groups, and one-way ANOVA tests for three or more groups (GraphPad Software, La Jolla, CA, USA). Clinical characteristics were analyzed using Chi-squared test. The correlation between overall survival (OS) and HMGB3 expression was performed using the Kaplan–Meier survival analysis and the log-rank test. *P* < 0.05 was considered significant.

## Results

### High HMGB3 expression indicates poor prognosis and chemoresistance in HGSOC

**Fig. 1:**
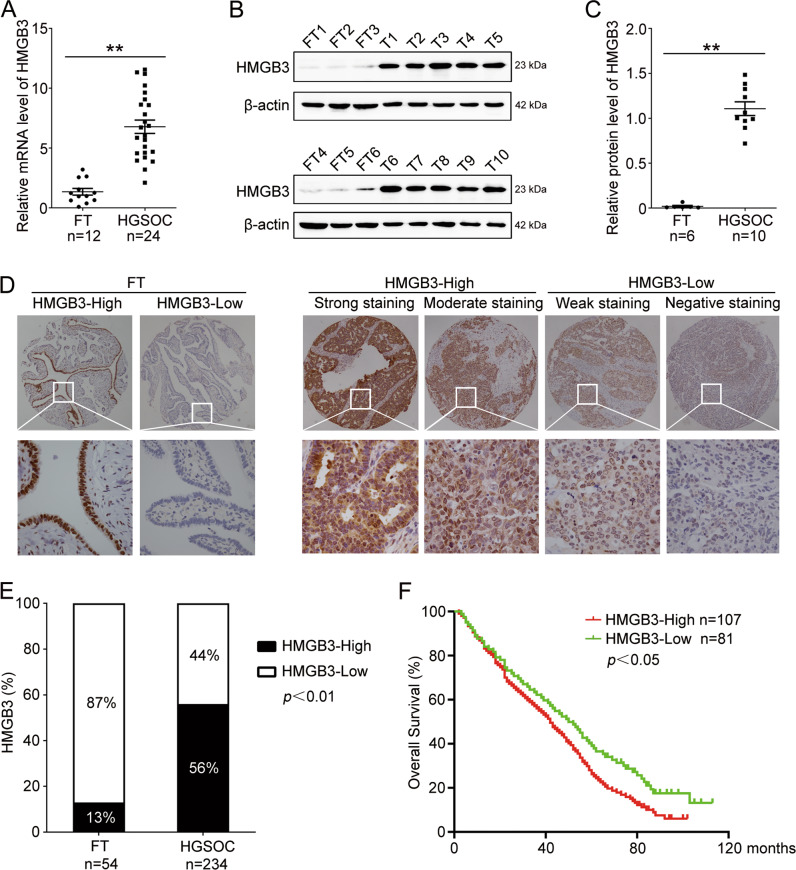
HMGB3 is highly expressed and correlated with poor prognosis in HGSOC. **A** qRT-PCR was performed to detect the mRNA levels of HMGB3 in high-grade serous ovarian cancer (HGSOC) tissues and normal fallopian tube (FT) tissues (FT, *n* = 12; HGSOC, *n* = 24). **B** Western blot was used to determine the protein levels of HMGB3 in FT and HGSOC tissues (FT, FT1-FT6; HGSOC, T1-T10). **C** Quantification of HMGB3 protein levels in (**B**). **D** Immunohistochemistry (IHC) staining of HMGB3 in FT tissues and HGSOC tissues. Representative images are shown (Upper, 40×; Lower, 400×). **E** Quantification of HMGB3 expression in FT tissues (*n* = 54) and HGSOC tissues (*n* = 234). **F** Overall survival (OS) curves of HGSOC patients with high or low HMGB3 levels. (Data are presented as the mean ± SEM, ^#^*p* > 0.05, *p < 0.05, **p < 0.01).

First, we investigated the expression and clinicopathological characteristics of HMGB3 in HGSOC tissues and normal FT tissues. Data from The Cancer Genome Atlas (TCGA) showed that HMGB3 was aberrantly expressed in a broad range of human cancer tissues, including ovarian cancer (Fig. S1A). Several sequencing results from different research groups have demonstrated that HMGB3 is overexpressed in ovarian cancer tissues compared with control tissues (Fig. S1B–F). Consistently, our results also showed that the HMGB3 mRNA and protein levels in HGSOC tissues were extremely upregulated relative to those in normal FT tissues (Fig. 1A–C). To explore the clinical significance of HMGB3 in patients with HGSOC, IHC staining was performed in HGSOC and FT tissues. HMGB3 was highly expressed in HGSOC tissues compared with normal FT tissues, with 56% of HGSOC samples classified as having a high expression rate (HMGB3-High) compared with only 13% of FT samples (Fig. 1D, E). Patients with HMGB3-High showed significantly shorter OS than patients with low HMGB3 expression (Fig. 1F). Moreover, the clinicopathological characteristic analysis found that HMGB3-High was associated with lymph node metastasis and platinum resistance in HGSOC patients (Table 1), indicating that HMGB3 might play a vital role in the development of chemoresistance in ovarian cancer.

### HMGB3 promotes olaparib resistance in ovarian cancer cells

**Fig. 2:**
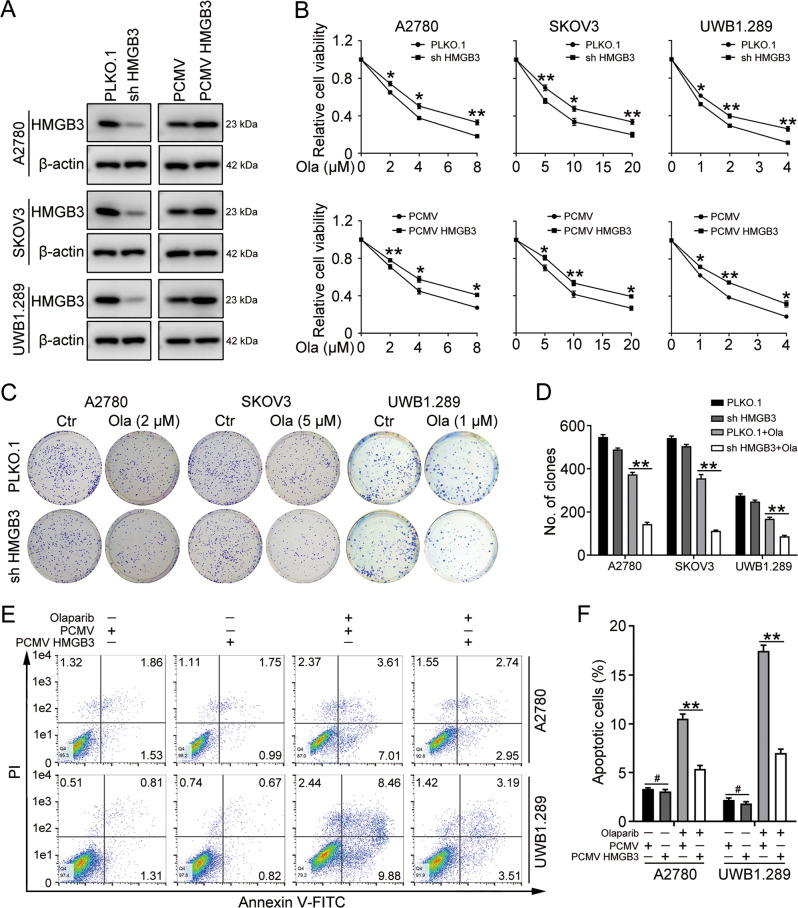
HMGB3 promotes olaparib resistance in ovarian cancer. **A** PLKO.1, HMGB3 shRNA (shHMGB3), PCMV, and PCMV HMGB3 plasmids were stably transfected into A2780, SKOV3, and UWB1.289 cells. Western blot was used to determine HMGB3 protein levels. Quantification of HMGB3 protein levels can be found in Fig. S2. **B** The MTT assay was performed to detect cell viability in A2780, SKOV3, and UWB1.289 cells treated with olaparib (Ola) for 72 h. **C** Clonogenic assay was conducted to assess the colony formation efficiency of A2780, SKOV3, and UWB1.289 cells in the presence of olaparib for 7–14 days. **D** Quantification of the number of clones in (**C**). **E** Flow cytometry assay was performed to detect cell apoptosis in A2780 and UWB1.289 cells treated with olaparib (A2780, 4 µM; UWB1.289, 2 µM) for 72 h. **F** Quantification of the proportion of apoptotic cells in (**E**). (Data are presented as the mean ± SEM, ^#^*p* > 0.05, *p < 0.05, **p < 0.01, *n* = 3).

**Fig. 3:**
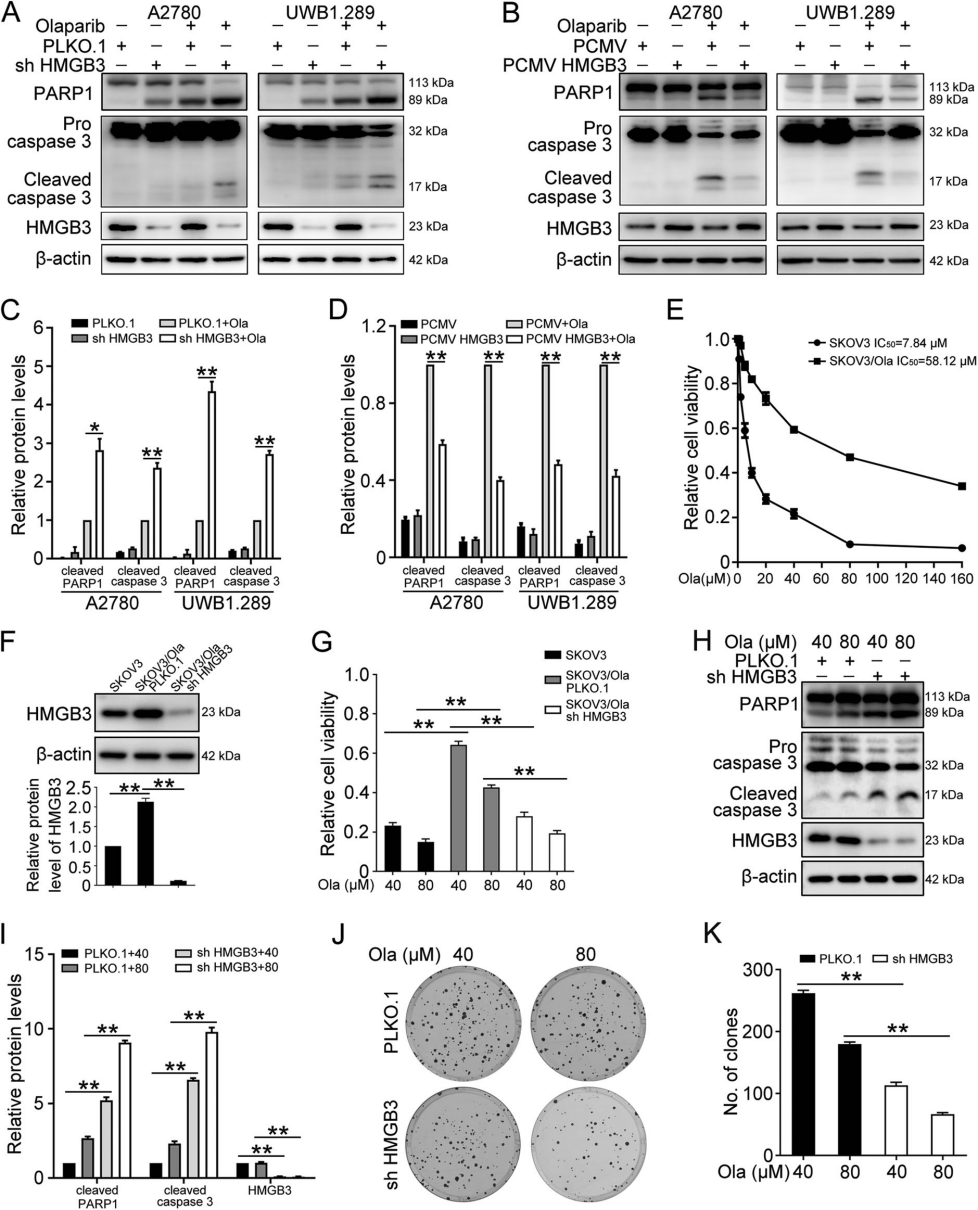
HMGB3 increases olaparib insensitivity in ovarian cancer cells. **A**, **B** A2780 and UWB1.289 cells transfected with PLKO.1, HMGB3 shRNA (shHMGB3), PCMV, and PCMV HMGB3 were treated with olaparib (A2780, 4 µM; UWB1.289, 2 µM) for 72 h. Western blot was conducted to detect the protein levels of PARP1, caspase 3, HMGB3, and β-actin. **C**, **D** Quantification of the protein levels of cleaved PARP1, cleaved caspase 3, and HMGB3 in (**A**) and (**B**), respectively. **E** SKOV3 and SKOV3/Ola cells were treated with different concentrations of olaparib (Ola; 0, 1, 2, 5, 10, 20, 40, 80, 160 μM) for 72 h. The MTT assay was used to determine cell viability. **F** Olaparib-resistant SKOV3 (SKOV3/Ola) were stably transfected with PLKO.1 or HMGB3 shRNA (sh HMGB3). Western blot was conducted to detect the protein levels of HMGB3 and β-actin in SKOV3 and SKOV3/Ola cells. **G** SKOV3 and SKOV3/Ola cells were treated with 40 or 80 μM olaparib for 72 h. The MTT assay was used to determine the cell viability. **H** Western blot was used to detect the protein levels of PARP1, caspase-3, HMGB3, and β-actin. **I** Quantification of the protein levels in (**H**). **J** SKOV3/Ola cells transfected with PLKO.1 or HMGB3 shRNA (sh HMGB3) were treated with 40 or 80 μM olaparib for 7–14 days. Clonogenic assay was conducted to assess the colony formation efficiency. **K** Quantification of the number of clones in (**J**). (Data are presented as the mean ± SEM, *p < 0.05, **p < 0.01, *n* = 3).

To generate stable cell lines with HMGB3 knockdown or overexpression, PLKO.1, HMGB3 shRNA (shHMGB3), PCMV, and PCMV HMGB3 plasmids were transfected into A2780, SKOV3, and UWB1.289 cells using a lentiviral vector. Western blot was performed to confirm the overexpression or knockdown efficiency of HMGB3 in these cells (Fig. 2A). The knockdown of HMGB3 significantly enhanced the sensitivity of A2780, SKOV3, and UWB1.289 cells to olaparib. By contrast, cells with HMGB3 overexpression showed increased cell viability following olaparib treatment (Fig. 2B). Correspondingly, cells with HMGB3 knockdown exhibited a decreased clonogenic ability in response to olaparib treatment (Fig. 2C, D). The flow cytometry assay verified that the overexpression of HMGB3 dramatically decreased the proportion of apoptotic cells following olaparib treatment (Fig. 2E, F). Besides, the overexpression of HMGB3 significantly reduced the protein levels of cleaved caspase 3 and cleaved PARP1 following olaparib treatment, whereas ovarian cancer cells with HMGB3 knockdown showed increased levels of cleaved caspase 3 and cleaved PARP1 (Fig. 3A–D).

Next, we investigated the PARPi resistance function of HMGB3 in olaparib-resistant SKOV3 (SKOV3/Ola) cells. The half-maximal inhibitory concentration (IC_50_) value for olaparib treatment in SKOV3/Ola cells was determined to be 58.12 µM, compared with 7.84 µM in the original parent SKOV3 cell line (Fig. 3E). The protein level of HMGB3 in SKOV3/Ola cells was significantly higher than that of the parent SKOV3 cells (Fig. 3F). The MTT results showed that HMGB3 knockdown significantly reversed olaparib resistance in SKOV3/Ola cells (Fig. 3F, G). In addition, olaparib-induced apoptosis was significantly enhanced in SKOV3/Ola cells following HMGB3 knockdown compared with control cells (Fig. 3H, I). HMGB3 knockdown also decreased the colony-forming abilities of SKOV3/Ola cells in response to olaparib (Fig. 3J, K). These results indicated that the upregulated expression of HMGB3 contributed to PARPi resistance in ovarian cancer cells.

### PARP1 is a direct interaction partner of HMGB3

**Fig. 4:**
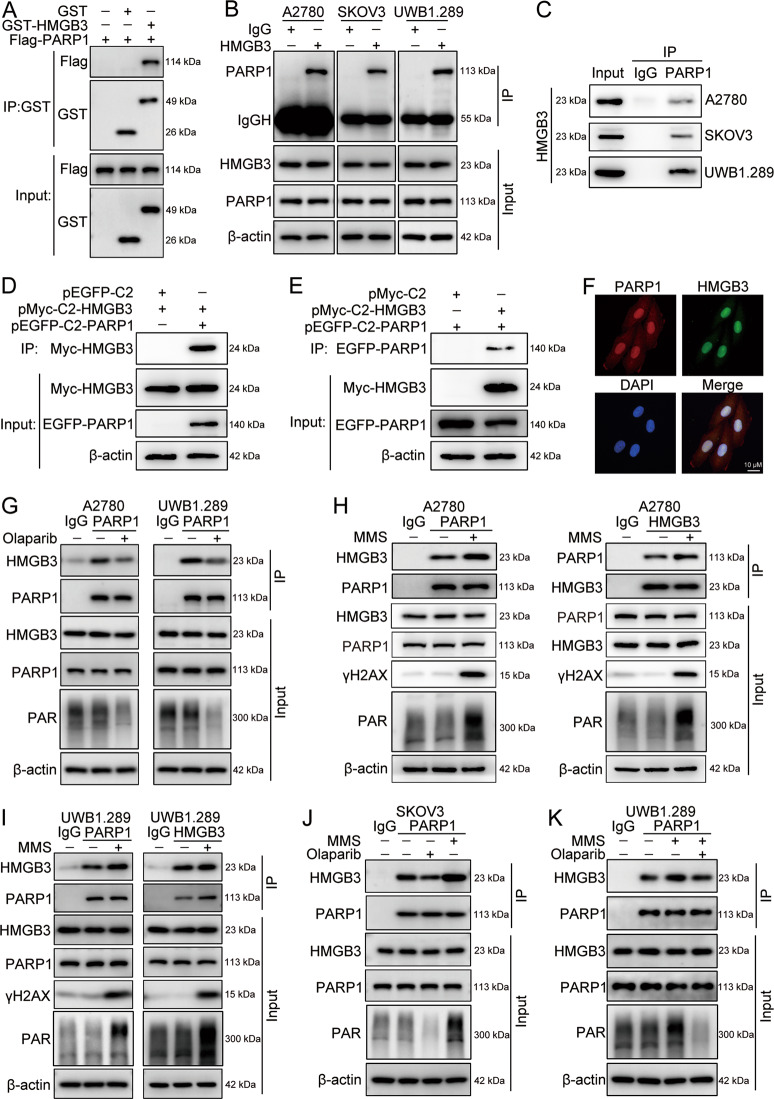
HMGB3 directly interacts with PARP1. **A** GST pull-down assay was used to verify the direct interaction between HMGB3 and PARP1. **B**, **C** Co-IP was performed to verify the endogenous interaction between HMGB3 and PARP1 in A2780, SKOV3, and UWB1.289 cells. **D**, **E** HEK293T cells were co-transfected with pMyc-C2-HMGB3 and pEGFP-C2-PARP1 plasmids for 48 h. Western blot analysis of exogenous EGFP-PARP1 or Myc-HMGB3 proteins co-immunoprecipitated (Co-IPed) with anti-Myc or anti-GFP antibody, respectively. **F** Double immunofluorescence staining showed the intracellular localization of HMGB3 (green) and PARP1 (red) in UWB1.289 cells. The nucleus was stained with DAPI (blue). Scale bar: 10 µm. **G** A2780 and UWB1.289 cells were treated with olaparib (A2780, 4 µM; UWB1.289, 2 µM) for 12 h. Co-IP was performed using anti-PARP1 antibody or mouse IgG isotype control. Western blot was used to detect HMGB3 and PARP1 from precipitated proteins. HMGB3, PARP1, PAR, and β-actin were detected among the input proteins. **H**, **I** A2780 and UWB1.289 cells were treated with 4 mM MMS for 20 min. Co-IP was performed using an anti-PARP1 antibody, anti-HMGB3 antibody, or IgG isotype control. Western blot was used to detect PARP1 and HMGB3 among the precipitated proteins. PARP1, HMGB3, γH2AX, PAR, and β-actin were detected among the input proteins. **J** SKOV3 cells were treated with olaparib (10 µM) for 12 h or MMS (4 mM) for 20 min. Co-IP and western blot were performed to determine the interaction between HMGB3 and PARP1. **K** UWB1.289 cells were treated with olaparib (2 µM) for 4 h prior to incubation with MMS (4 mM) for 20 min. Co-IP and western blot were performed to determine the interaction between HMGB3 and PARP1. (*n* = 3). Quantification of the protein levels can be found in Fig. S4.

To determine which protein interact with HMGB3 in response to PARPi treatment, MS-based proteomic analysis was performed. Remarkably, PARP1 was the most abundant protein among the identified HMGB3 binding partners. GST pull-down assay verified the direct interaction between HMGB3 and PARP1 (Fig. 4A). To characterize the interaction between endogenous HMGB3 and PARP1, A2780, SKOV3, and UWB1.289 cell lysates were collected, and Co-IP assays were performed using control IgG, anti-HMGB3, or anti-PARP1 antibody. PARP1 was clearly detected in precipitations pulled down by anti-HMGB3. Consistently, HMGB3 was detected in the immunoprecipitated complex obtained using anti-PARP1 (Fig. 4B, C). To further confirm the interaction between HMGB3 and PARP1, exogenous pMyc-C2-HMGB3 and pEGFP-C2-PARP1 plasmids were transiently co-transfected into HEK293T cells, and Co-IP experiments were conducted. As shown in Fig. 4D, E, EGFP-PARP1 or Myc-HMGB3 were detected among the proteins precipitated by anti-Myc or anti-GFP antibodies, respectively. Additionally, double immunofluorescence staining demonstrated that both PARP1 and HMGB3 proteins localize in the nucleus in UWB1.289 cells (Fig. 4F).

PARP1 plays a crucial role in DNA repair pathways through its poly(ADP-ribosylation) (PARylation) activity, which utilizes nicotinamide adenine dinucleotide (NAD^+^) to form PAR polymers that are then transferred to acceptor proteins, including PARP1 itself [24, 25]. Olaparib has been shown to suppress PARP1 catalytic activity through PARP trapping, which ultimately leads to DNA damage and cell death [26]. We investigated the influence of PARylation on the interaction between HMGB3 and PARP1. Co-IP experiments, followed by western blot analysis, revealed that olaparib treatment restrained the physical interaction between HMGB3 and PARP1 in A2780 and UWB1.289 cells (Fig. 4G). MMS, a well-known DNA alkylating agent, can be used to induce DNA damage and trigger a strong protein PARylation response [27]. DNA damage caused by MMS significantly promoted the interaction between HMGB3 and PARP1 in A2780 and UWB1.289 cells (Fig. 4H, I). The effects of olaparib and MMS on the interaction between HMGB3 and PARP1 were further verified in SKOV3 cells (Fig. 4J). Besides, the MMS-enhanced interaction between HMGB3 and PARP1 was restrained by olaparib (Fig. 4K). These results suggested that PARP1 directly interacts with HMGB3 and that this interaction may rely on the PARylation activity of PARP1.

### HMGB3 facilitates the PARylation activity of PARP1

**Fig. 5:**
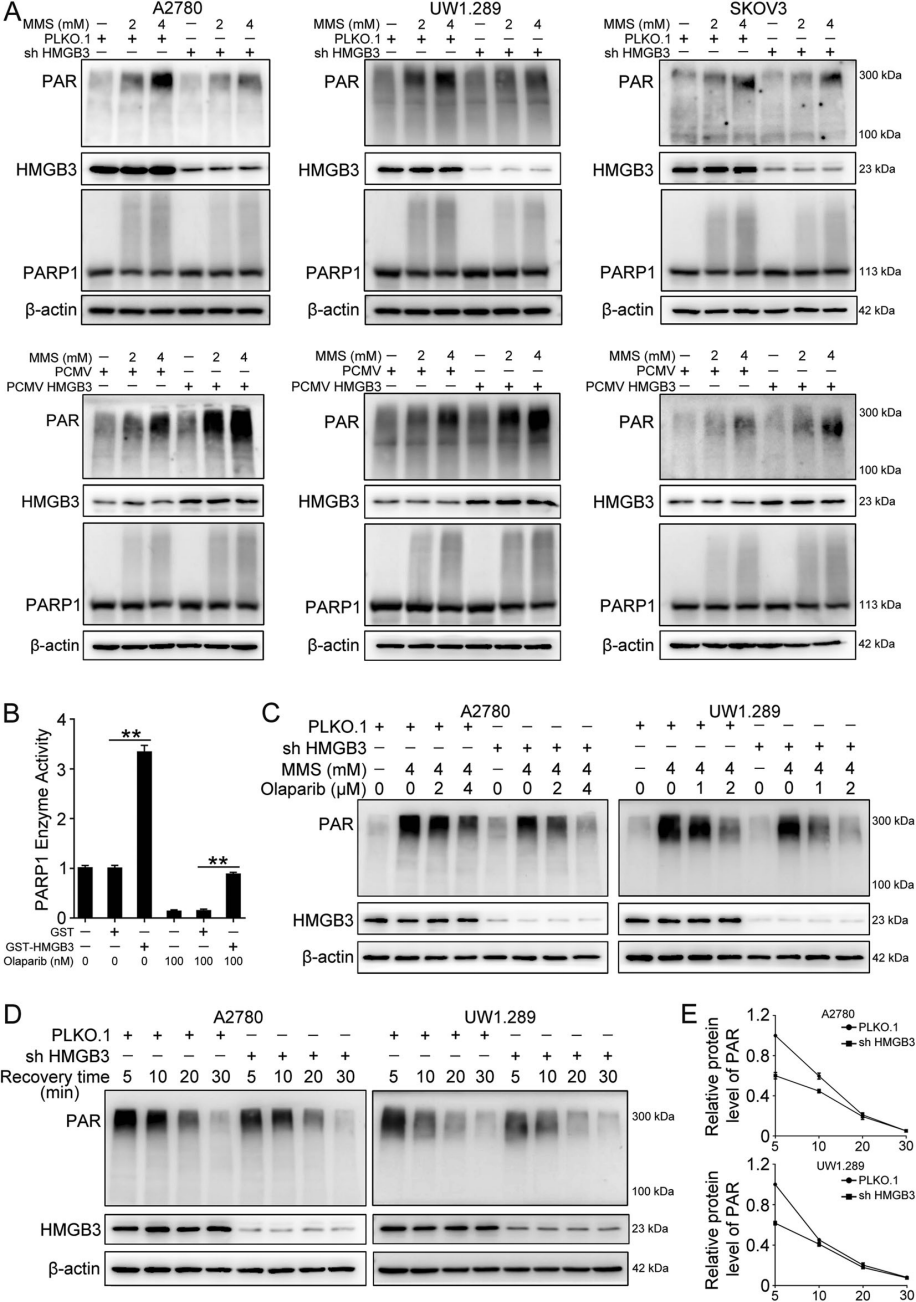
HMGB3 enhances DNA damage-induced PARP1 activity (PARylation). **A** A2780, UWB1.289, and SKOV3 cells transfected with PLKO.1, HMGB3 shRNA (shHMGB3), PCMV, and PCMV HMGB3 were treated with 2 mM or 4 mM MMS for 20 min. Western blot was performed to detect the protein levels of PAR, HMGB3, PARP1, and β-actin. Quantification of the PAR protein levels can be found in Fig. S5A. **B** Activity of human recombinant PARP1 in the presence or absence of olaparib. **C** A2780 and UWB1.289 cells transfected with PLKO.1 and HMGB3 shRNA (shHMGB3) were treated with different concentrations of olaparib for 4 h prior to incubation with 4 mM MMS for 20 min. Western blot was conducted to detect the protein levels of PAR, HMGB3, and β-actin. Quantification of the PAR protein levels can be found in Fig. S5B. **D** A2780 and UWB1.289 cells transfected with PLKO.1 and HMGB3 shRNA (shHMGB3) were treated with 4 mM MMS for 20 min. The culture medium containing MMS was replaced with normal culture medium, and cell lysates were collected at defined time points (5, 10, 20, and 30 min). Western blot was used to detect the protein levels of PAR, HMGB3, and β-actin. **E** Quantification of the PAR protein levels in (**D**). (Data are presented as the mean ± SEM, *p < 0.05, **p < 0.01, *n* = 3).

To study the effects of HMGB3 on the PARylation activity of PARP1, ovarian cancer cells were exposed to MMS to induce a DNA damage response in the presence of HMGB3 knockdown or overexpression. Western blot results showed that HMGB3 knockdown dramatically reduced the level of total PARylated cellular proteins in A2780, SKOV3, and UWB1.289 cells following MMS treatment, whereas the overexpression of HMGB3 resulted in the apparent increase in PARylated proteins compared to PCMV controls (Fig. 5A). To further confirm the influence of HMGB3 on PARP1 activity, an in vitro assay with recombinant PARP1 and HMGB3 was performed. The in vitro assay demonstrated that the addition of HMGB3 increases the PARP1 enzyme activity. Upon olaparib treatment, the activity of PARP1 was significantly higher in the presence of HMGB3 protein compared to that in the control group (Fig. 5B). Next, we investigated the influence of increasing concentrations of olaparib on PARylation levels in cells with HMGB3 knockdown or not. Upon MMS challenge, a relatively lower concentration of olaparib was required to decrease PARylation levels in cells with HMGB3 knockdown (Fig. 5C). The observed reduction in PARylated proteins following HMGB3 knockdown could be due to either decreased PARylation activity or increased dePARylation activity. To determine which pathway was affected, A2780 and UWB1.289 cells were transfected with PLKO.1 or HMGB3 shRNA (sh HMGB3) and collected at different time points following MMS treatment. The PARylated proteins were detected during the recovery period. Western blot analysis revealed that although cells transfected with PLKO.1 showed higher PARylation levels immediately following MMS treatment, the levels of PARylated proteins became almost undetectable in both groups within 30 min after MMS treatment (Fig. 5D, E). These data revealed a newly discovered function for HMGB3 in the facilitation of protein PARylation in ovarian cancer cells.

### HMGB3 inhibits PARP trapping and DNA damage induced by olaparib

**Fig. 6:**
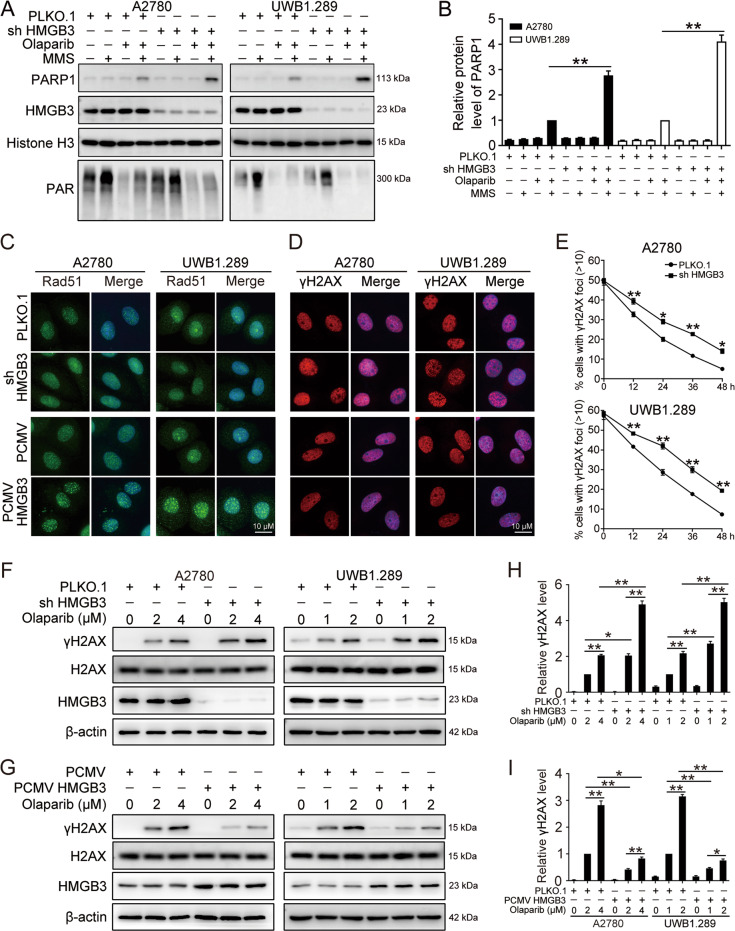
HMGB3 prevents PARP1 trapping and confers DNA repair upon olaparib treatment. **A** A2780 and UWB1.289 cells transfected with PLKO.1 and HMGB3 shRNA (shHMGB3) were treated with olaparib (A2780, 4 µM; UWB1.289, 2 µM) for 4 h prior to incubation with 4 mM MMS for 20 min. Western blot was conducted to detect the protein levels of PARP1 in the chromatin-bound fractions. **B** Quantification of the PARP1 protein levels in (**A**). **C**, **D** A2780 and UWB1.289 cells transfected with PLKO.1, HMGB3 shRNA (shHMGB3), PCMV, and PCMV HMGB3 were treated with olaparib (A2780, 4 µM; UWB1.289, 2 µM) for 72 h. Rad51 and γH2AX foci were examined by immunofluorescence staining. Scale bar: 10 µm. Quantification of the number of γH2AX and RAD51 foci (>10) can be found in Fig. S6B. **E** A2780 and UWB1.289 cells transfected with PLKO.1 and HMGB3 shRNA (shHMGB3) were treated with olaparib (A2780, 10 µM; UWB1.289, 5 µM) for 24 h. After that, olaparib was removed and fresh drug free media was added. Cells were cultured further and γH2AX foci were observed at 12 h, 24 h, 36 h, and 48 h post drug removal. **F**, **G** A2780 and UWB1.289 cells transfected with PLKO.1, HMGB3 shRNA (shHMGB3), PCMV, and PCMV HMGB3 were treated with olaparib (A2780, 2 µM and 4 µM; UWB1.289, 1 µM and 2 µM) for 72 h. The protein level of γH2AX was detected by western blot. **H**, **I** Quantification of the protein level of γH2AX in (**F**) and (**G**), respectively. (Data are presented as the mean ± SEM, *p < 0.05, **p < 0.01, *n* = 3).

To further determine the role played by HMGB3 in the regulation of PAPR1 DNA trapping induced by olaparib treatment, A2780 and UWB1.289 cells transfected with PLKO.1 or HMGB3 shRNA (sh HMGB3) were exposed to olaparib with or without MMS. Chromatin-bound proteins were extracted and analyzed using western blot. Our results indicated that HMGB3 knockdown dramatically increased PARP1 levels among chromatin-bound proteins, indicating that HMGB3 prevented the olaparib-induced PAPR1 trapping at DNA damage sites (Fig. 6A, B). In addition, the overexpression of HMGB3 led to an increase in Rad51 foci accumulation and a reduction in γH2AX foci accumulation compared with the control group following olaparib treatment, suggesting an upregulation of the DNA repair capability. By contrast, HMGB3 knockdown decreased Rad51 accumulation and increased γH2AX accumulation (Fig. 6C, D). DNA repair kinetics assay was also performed for 12, 24, 36, and 48 h after treatment with olaparib for 24 h. Cells with HMGB3 knockdown showed a significant delay in resolution of γH2AX foci compared with the control group, indicative of defective repair in the HMGB3 deficient setting (Fig. 6E). Consistent with these findings, western blot results showed that the protein levels of γH2AX in cells with HMGB3 knockdown were significantly increased compared with those in the control group in response to olaparib, whereas the overexpression of HMGB3 had the opposite effect (Fig. 6F–I). Thus, HMGB3 served as a positive factor in the regulation of the PARP1-mediated DNA damage response.

### HMGB3 promotes olaparib resistance in vivo

**Fig. 7:**
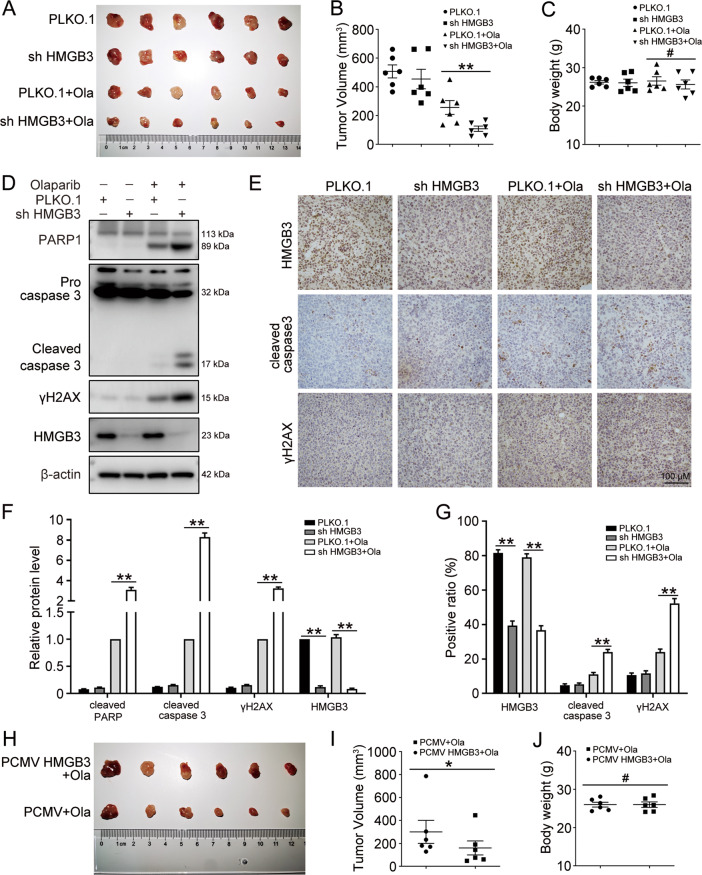
HMGB3 promotes olaparib resistance in the xenograft mouse model. UWB1.289 cells (5 × 10^6^ cells) transfected with PLKO.1 and HMGB3 shRNA (shHMGB3) were subcutaneously injected into the left armpit of each mouse. When the tumor volumes reached ~50 mm^3^, the mice were evenly divided into four groups (PLKO.1, shHMGB3, PLKO.1 + Ola, shHMGB3 + Ola) and received an intraperitoneal injection of olaparib (Ola, 50 mg/kg) or PBS once a day. Twenty days post-injection, the mice were sacrificed, and mouse body weights and tumor volumes were quantified. **A** Tumors from each group are shown. **B** The tumor volumes of each group. **C** The mouse body weights of each group. **D** Western blot was used to detect the protein levels of PARP1, caspase 3, γH2AX, HMGB3, and β-actin in tumor tissues. **E** Representative images of IHC staining against HMGB3, cleaved caspase 3, and γH2AX in tumor tissues. Scale bar: 100 µm. **F** Quantification of relative protein expression levels in (**D**). **G** Quantification of the positive ratio of HMGB3, cleaved caspase 3, and γH2AX in (**E**). UWB1.289 cells (5 × 10^6^ cells) transfected with PCMV and PCMV HMGB3 were subcutaneously injected into the left armpit of each mouse. When the tumor volumes reached approximately 50 mm^3^, the mice were evenly divided into two groups (PCMV + Ola, PCMV HMGB3 + Ola) and received an intraperitoneal injection of olaparib (Ola, 50 mg/kg) or PBS once a day. Twenty days post-injection, the mice were sacrificed, and mouse body weights and tumor volumes were quantified. **H** Tumors from each group are shown. **I** The tumor volumes of each group. **J** The mouse body weights of each group. (Data are presented as the mean ± SEM, ^#^*p* > 0.05, *p < 0.05, **p < 0.01, *n* = 6).

Next, we assessed whether aberrant HMGB3 expression promoted the tumor response to olaparib treatment in a xenograft mouse model. BALB/c nude mice were subcutaneously injected with UWB1.289 cells transfected with PLKO.1 or HMGB3 shRNA (shHMGB3). Approximately one week later, the mice were equally divided into four groups and intraperitoneally injected with 50 mg/kg olaparib for 20 days. The tumor volumes in the PLKO.1 group were significantly higher than those in the shHMGB3 group, demonstrating the chemoresistant role of HMGB3 in vivo (Fig. 7A, B). Olaparib treatment or HMGB3 knockdown had no effects on the mouse body weight (Fig. 7C). Additionally, HMGB3 knockdown increased the protein level of γH2AX in response to olaparib in the xenograft model. The apoptotic level in the shHMGB3 group was significantly increased compared with that in PLKO.1 control group (Fig. 7D, F). IHC staining for HMGB3, cleaved caspase 3, and γH2AX verified that HMGB3 knockdown promoted cell apoptosis and the DNA damage response in ovarian cancer with olaparib treatment (Fig. 7E, G). Moreover, upon olaparib treatment, the tumor volumes were significantly higher in mice injected with PCMV HMGB3 cells than in those injected with PCMV cells (Fig. 7H–J). Therefore, our findings suggested that HMGB3 could confer PARPi resistance in vivo.

## Discussion

The abnormal expression of HMGB3 is thought to be correlated with an increased proliferative rate, increased metastatic ability, and poor OS in several tumor types [8]. HMGB3 deletion has been demonstrated to increase the cell sensitivity to cisplatin via the attenuation of ATR/CHK1/p-CHK1 DNA damage signaling pathway activation in ovarian cancer [22], suggesting an important role for HMGB3 in ovarian cancer. Here, we found that HMGB3 was significantly overexpressed in HGSOC tissues compared with FT control tissues, and high HMGB3 expression levels indicated shorter OS in patients with HGSOC. HMGB3 expression levels were also positively associated with lymph node metastasis and drug resistance. Our results suggested that HMGB3 possessed the potential to serve as a novel biomarker for tumor development and drug resistance in ovarian cancer.

The best-studied and most abundant member of the PARP protein family is PARP1, which mediates DNA repair through catalytic PARylation activity and accounts for 80–90% of all PARylation activity in cells [28]. PARP activity inhibition is synthetically lethal in cells with HR deficiency, especially in those with inactive BRCA1 and BRCA2 genes [29, 30]. Several PARPi have been approved for the clinical treatment of patients with BRCA1/2 mutations in pancreatic, prostate, ovarian, and breast cancers [31]. In recent years, PARPi have been reported to improve the clinical outcomes in patients regardless of the patients’ BRCA1/2 or HR-mediated DNA repair status [32, 33]. Although PARPi have greatly improved the outcomes of patients with ovarian cancer, especially among patients harboring BRCA1/2 mutations, the development of drug resistance remains a vexing challenge in most patients. Greater than 40% of ovarian cancer patients with BRCA1/2 deficiency fail to respond to PARPi [34]. In the current study, we reported for the first time that HMGB3 promoted PARPi resistance in both BRCA1-deficient and wild-type ovarian cancer cells. HMGB3 knockdown dramatically increased the sensitivity of ovarian cancer cells to PARPi, whereas HMGB3 overexpression facilitated the resistance of ovarian cancer cells to PARPi. Thus, the targeted deletion of HMGB3 might have a promising future in overcoming PARPi resistance during the clinical treatment of ovarian cancer. Many combination therapies have been proposed to overcome PARPi resistance and increase the drug efficiency of PARPi, such as the combination of olaparib with PI3K inhibitors, WEE1 kinase inhibitors, or topoisomerase 1 (TOP1) inhibitors [35]. Currently, no HMGB3 inhibitor has been developed, and we intend to develop HMGB3-specific inhibitors in future research to further verify the effects of HMGB3 inhibition in preclinical practice.

Thus far, the known mechanisms of PARPi resistance include the restoration of HR ability, increased drug efflux, diminished PARP1 trapping, and the stabilization of replication forks [36]. PARPi primarily act through the competitive binding of the NAD^+^ binding domain in PARP1, causing PARP1 to become trapped on DNA damaged sites, leading to the subsequent loss of the PARP1 PARylation activity [36]. HMGA2, another member of the HMG family, has been shown to prevent PARP1 chromatin trapping, increasing the PARylation activity of PARP1 through direct interaction with PARP1 [37]. HMG14 also interacts directly with PARP1 and promotes PARylation activity in vitro and in vivo [38]. Here, we identified HMGB3 as a novel interaction partner of PARP1. Further research showed that the DNA damage response induced by MMS promoted the interaction between HMGB3 and PARP1, whereas olaparib pre-treatment significantly inhibited this interaction. The overexpression of HMGB3 prevented PARP1 from becoming trapped at DNA lesion sites, resulting in the promotion of PARP1 PARylation activity and the inhibition of the DNA damage response induced by olaparib treatment. HMGB family members feature two conserved DNA-binding domains (A-box and B-box), which are capable of binding and bending DNA structures [8]; therefore, we speculate that the interaction between PARP1 and HMGB3 changes the PARP1 DNA-binding kinetics, further decreasing the ability of olaparib to trap PARP1 at DNA damage sites. Research is currently ongoing to better clarify the HMGB3 structural features that influence the binding of PARP1 with chromatin.

In the current study, we verified that HMGB3 was aberrantly overexpressed in HGSOC tissues, and high HMGB3 expression was an indicator of poor prognosis and drug resistance among patients with HGSOC. In addition, HMGB3 facilitated the development of PARPi resistance by directly interacting with PARP1 in ovarian cancer cells, and the inhibition of HMGB3 combined with PARPi might have broad prospects in the clinical therapy of ovarian cancer.

## Supplementary information

Supplementary information

Original Western Blots

Highlights

Conflicts of Interest Statement

Author contribution form

Checklist

## Data Availability

The datasets used and/or analyzed during the current study are available from the corresponding author on reasonable request.
