# Supplementary material for: HMGB3 promotes PARP inhibitor resistance through interacting with PARP1 in ovarian cancer
Source: Cell Death Dis. 2022 Mar 24;13(3):263. doi: 10.1038/s41419-022-04670-7 (PMC8948190; doi:10.1038/s41419-022-04670-7)
Supplement: Supplementary file 1 — Supplementary information [file 41419_2022_4670_MOESM1_ESM.pdf]

Supplementary Figures and Figure legends:

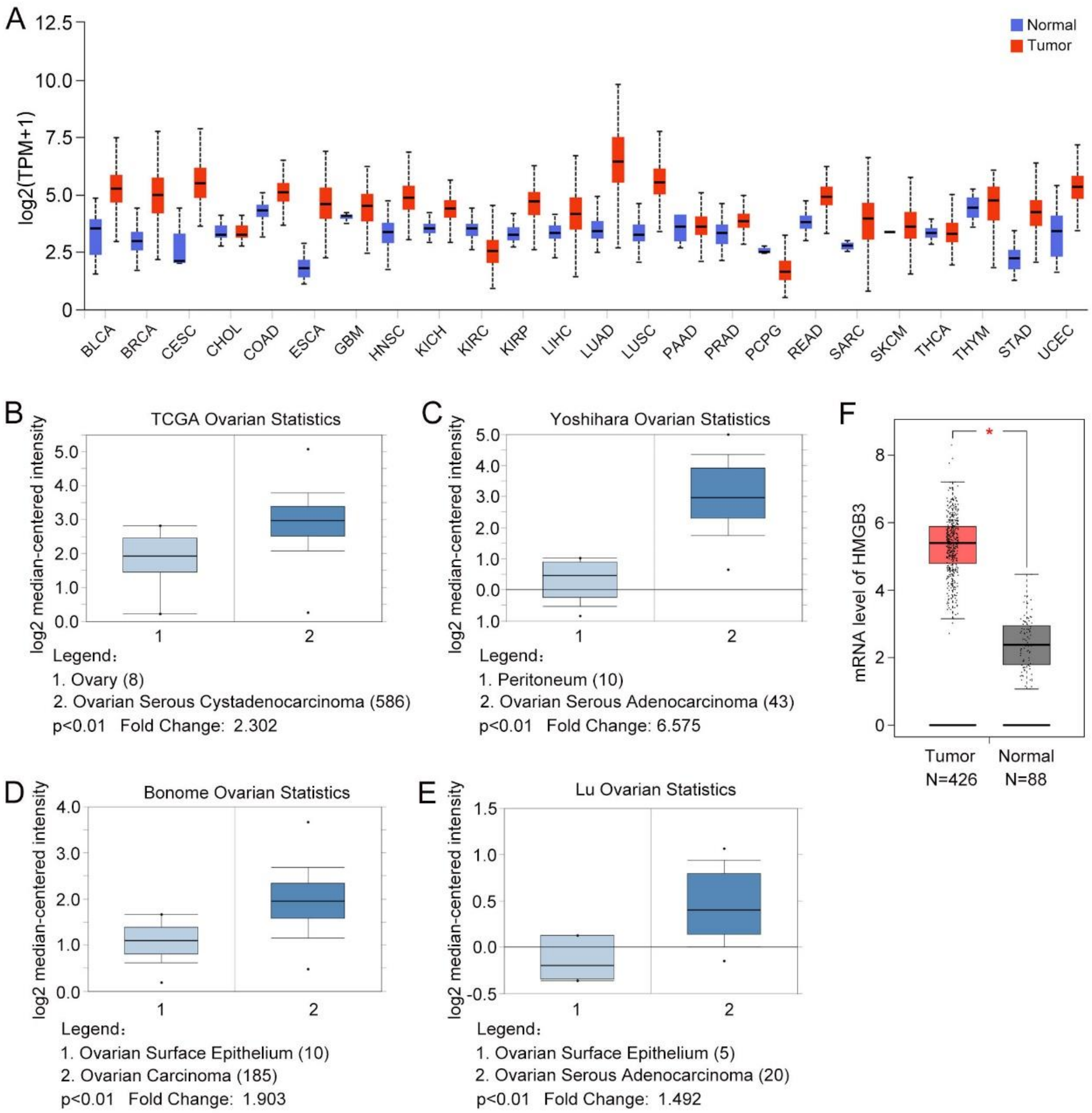

Figure S1. The expression of HMGB3 is extensively higher in ovarian cancer tissues. (A) TCGA dataset (http://ualcan.path.uab.edu/) showed that HMGB3 was highly expressed in a variety of cancer tissues. (B–E) Oncomine (https://www.oncomine.org) dataset showed that the expression of HMGB3 was relatively higher in ovarian cancer tissues compared with normal controls. (F) TCGA dataset (http://gepia.cancer-pku.cn) showed that HMGB3 was highly expressed in ovarian cancer tissues compared with normal controls. (Data are presented as the mean ± SEM, *$p < 0.05$).

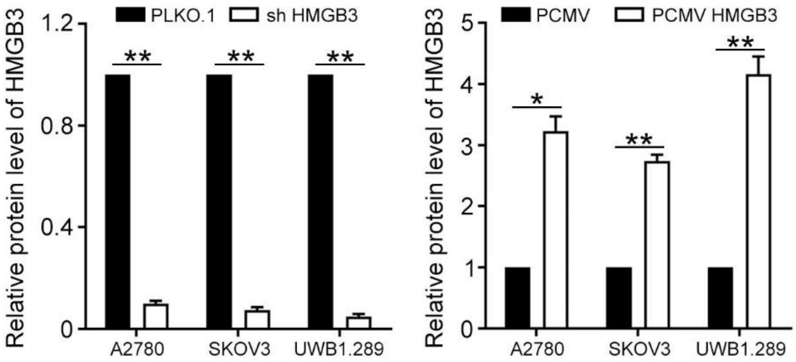

Figure S2. Quantification of HMGB3 protein levels in Figure 2A. (Data are presented as the mean ± SEM, *$p < 0.05$, **$p < 0.01$, n = 3).

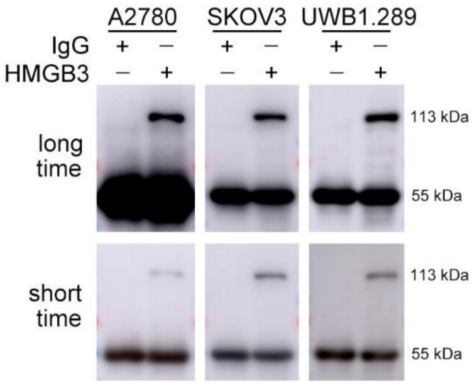

Figure S3. The blots of Figure 4B exposed in long and short time.

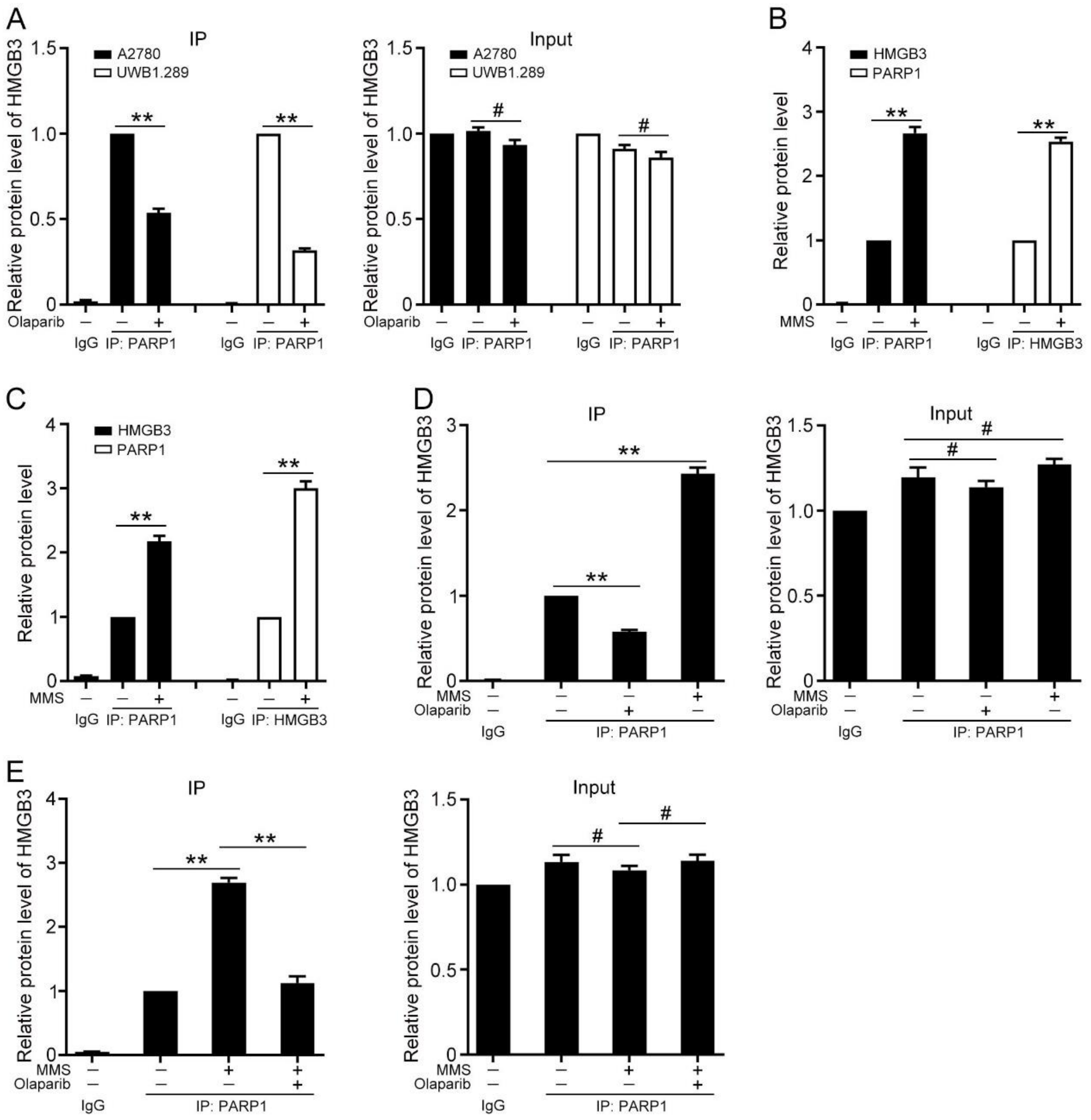

Figure S4. (A) Quantification of HMGB3 protein levels in Figure 4G. (B) Quantification of the protein levels of HMGB3 and PARP1 in Figure 4H. (C) Quantification of the protein levels of HMGB3 and PARP1 in Figure 4I. (D) Quantification of HMGB3 protein levels in Figure 4J. (E) Quantification of HMGB3 protein levels in Figure 4K. (Data are presented as the mean ± SEM, $^{\#}p > 0.05$, $^{*}p < 0.05$, $^{**}p < 0.01$, n = 3).

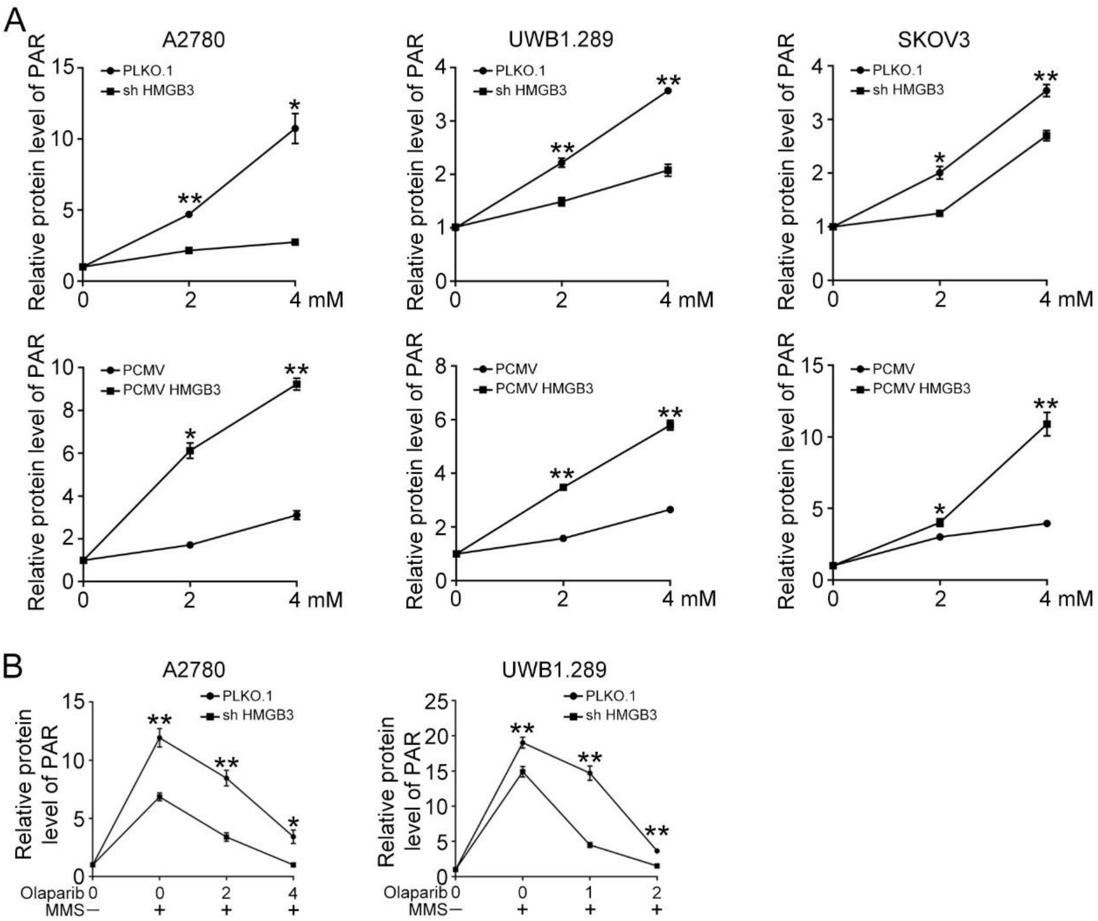

Figure S5. Quantification of PAR protein levels in Figure 5A. (B) Quantification of the PAR protein levels in Figure 5C. (Data are presented as the mean ± SEM, *p < 0.05, **p < 0.01, n = 3).

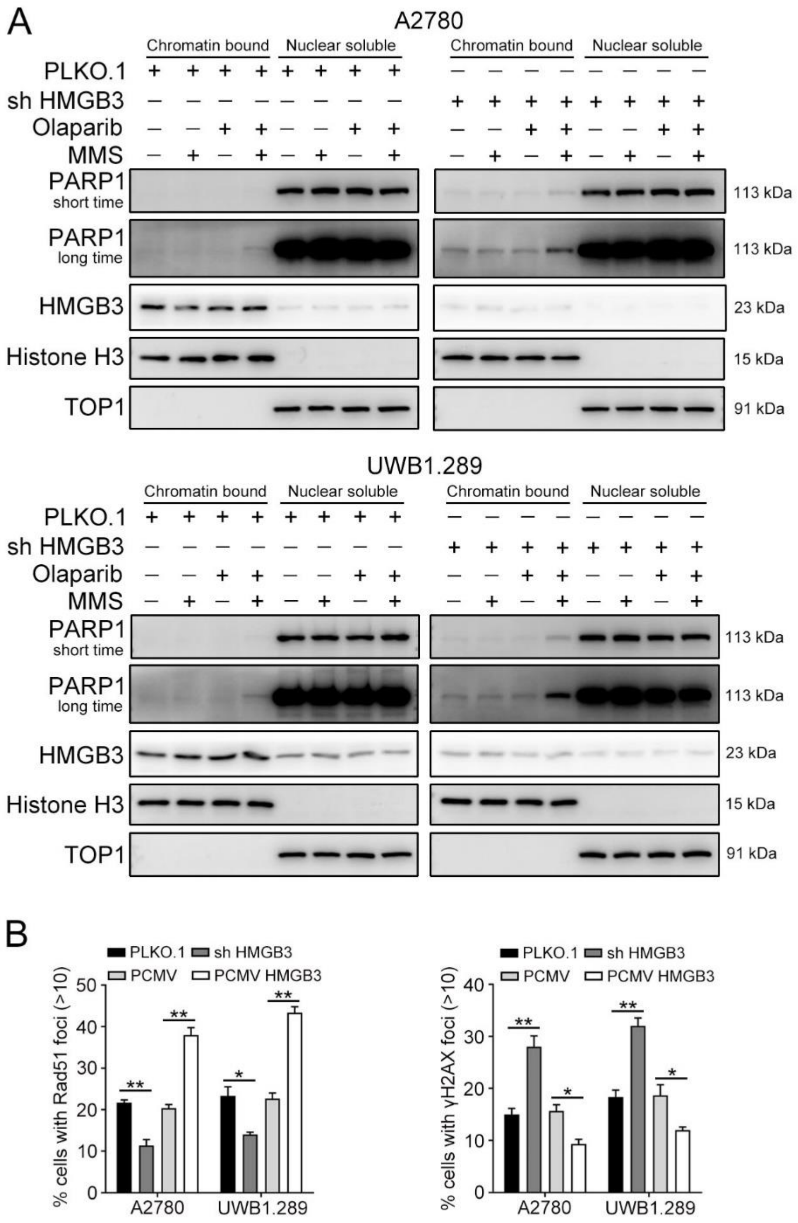

Figure S6. (A) A2780 and UWB1.289 cells transfected with PLKO.1 and HMGB3 shRNA (shHMGB3) were treated with olaparib (A2780, 4 μM; UWB1.289, 2 μM) for 4 h prior to incubation with 4 mM MMS for 20 min. Western blot was conducted to detect the protein levels of PARP1 in the chromatin-bound and nuclear soluble fractions. (B) Quantification of the number of γH2AX and RAD51 foci (>10) in Figure 6C and D. (Data are presented as the mean ± SEM, **p* < 0.05, ***p* < 0.01, n = 3).

Table S1. Primer sequences used for qRT-PCR

Gene	Primer sequences (5' to 3')
β-actin-F	CATGTACGTTGCTATCCAGGC
β-actin-R	CTCCTTAATGTCACGCACGAT
HMGB3-F	CCAAGAAGTGCTCTGAGAGGTG
HMGB3-R	CTTCTTGCCTCCCTTAGCTGGT
