# Supplementary figures and images for: HMGB3 promotes PARP inhibitor resistance through interacting with PARP1 in ovarian cancer
Source: Cell Death Dis. 2022 Mar 24;13(3):263. doi: 10.1038/s41419-022-04670-7 (PMC8948190; doi:10.1038/s41419-022-04670-7)

Figure 1

1B

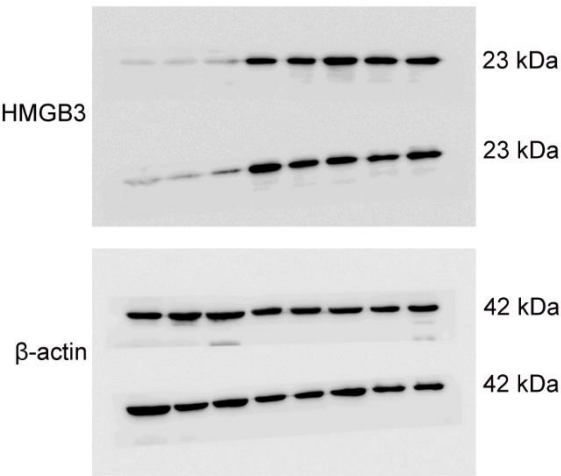

Figure 2

2A

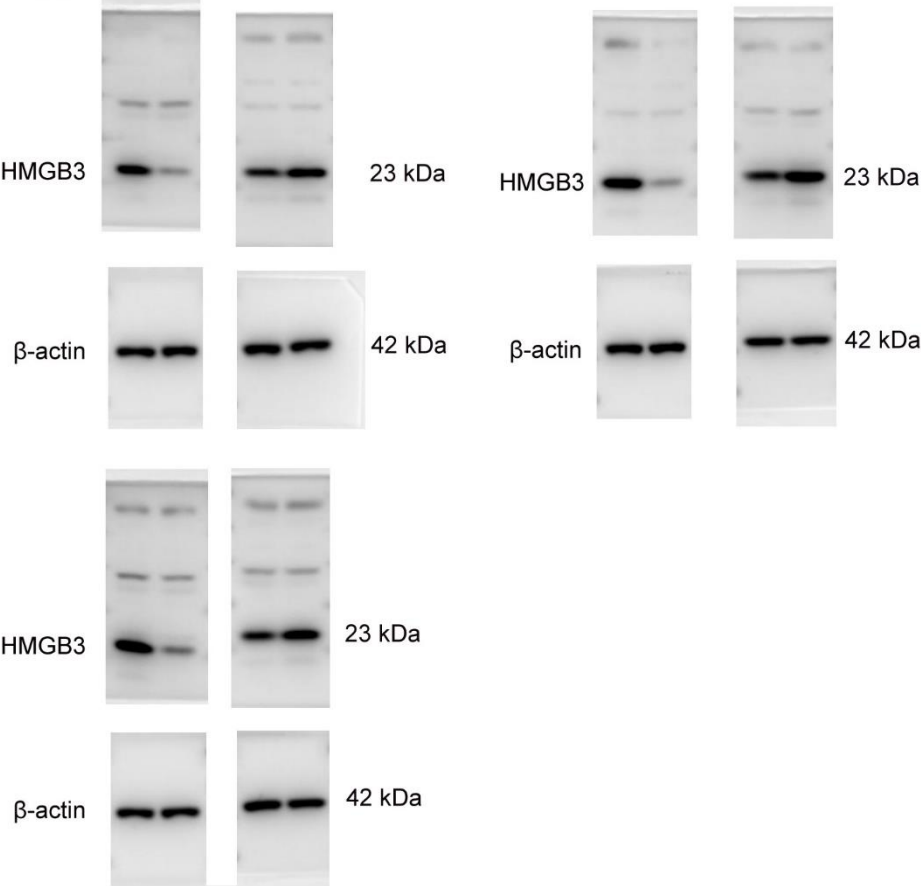

Figure 3

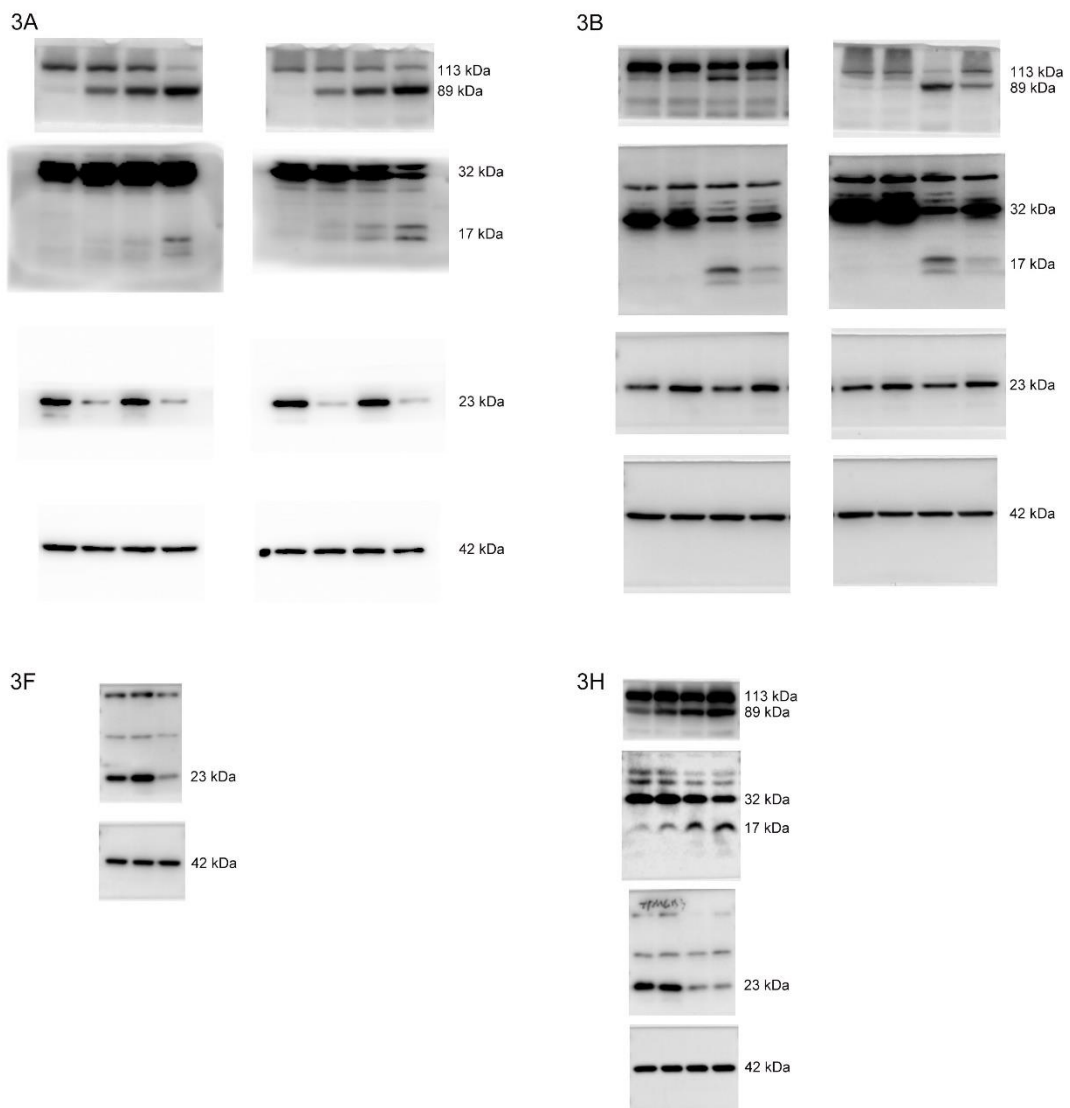

Figure 4

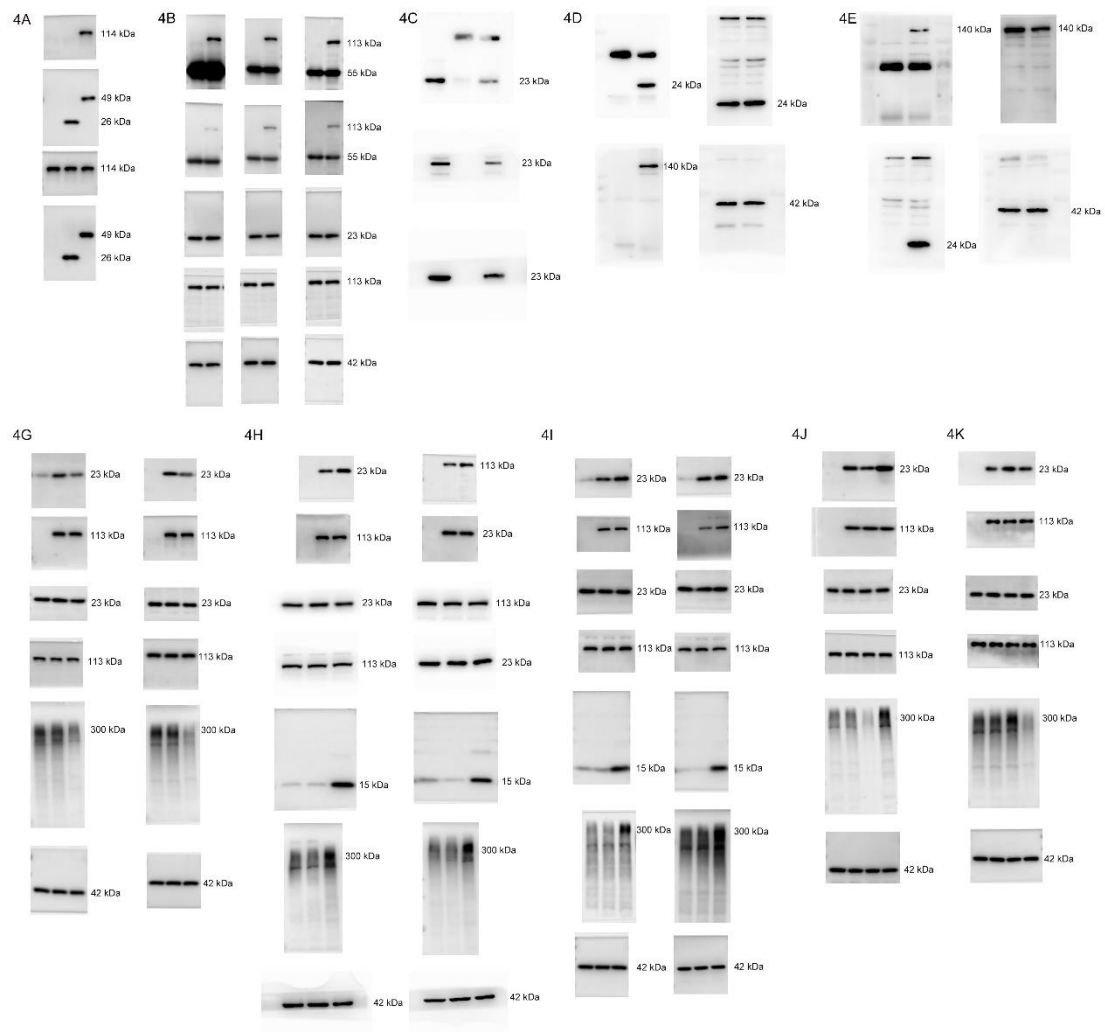

Figure 5

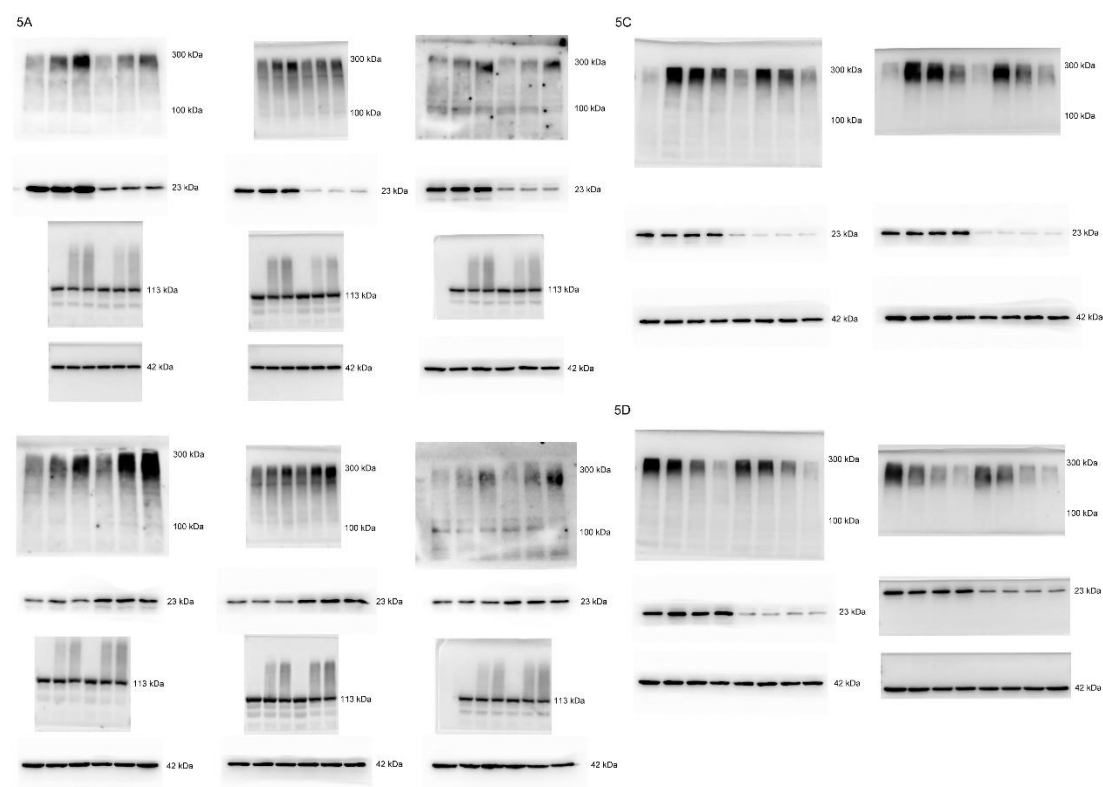

Figure 6

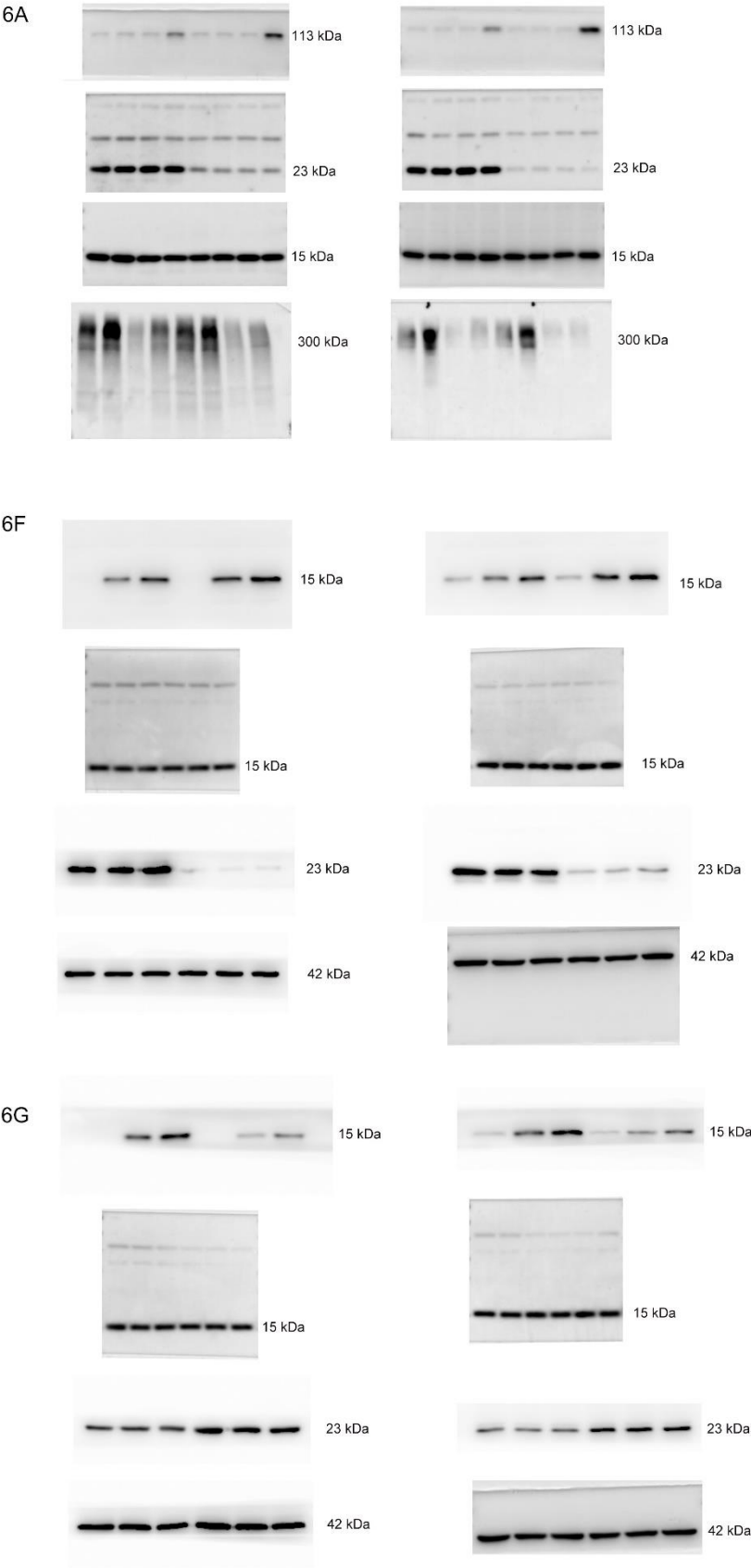

Figure 7

7D

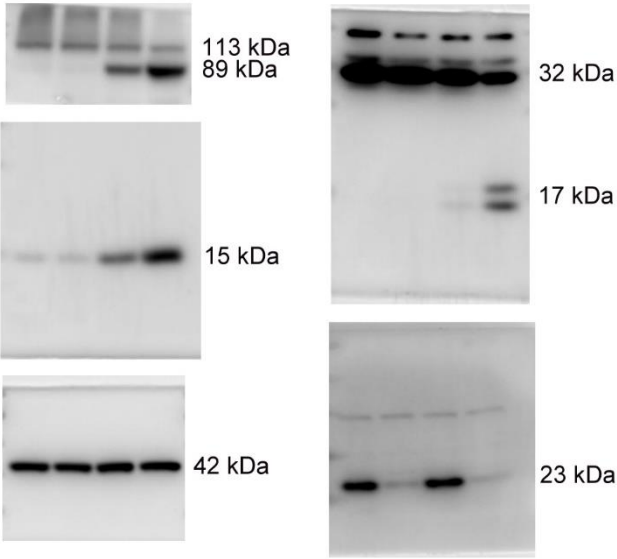

Supplementary Figure S6

S6A

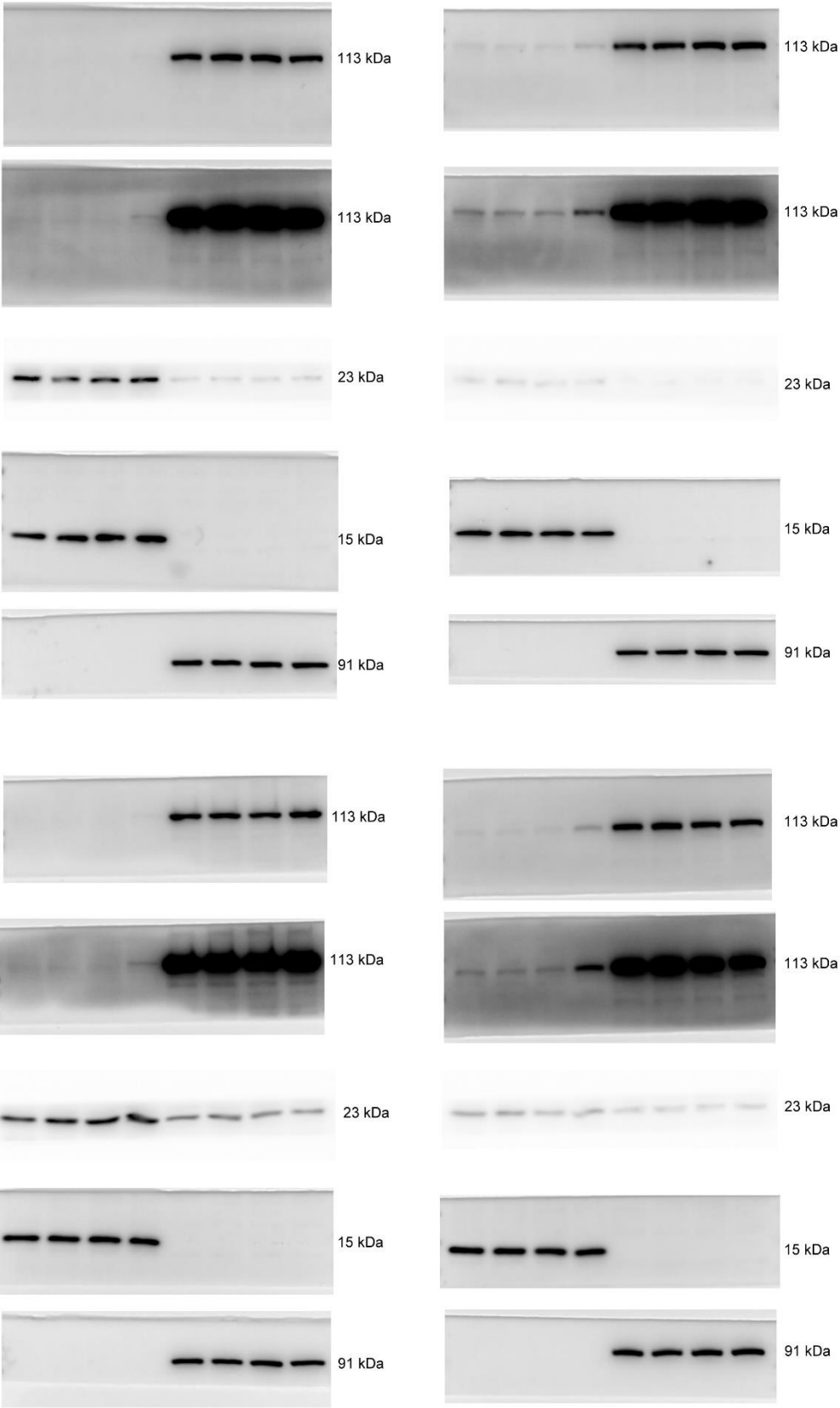

Supplement: Supplementary file 2 — Original Western Blots [file 41419_2022_4670_MOESM2_ESM.pdf]
