# Supplementary material for: HMGB3 promotes PARP inhibitor resistance through interacting with PARP1 in ovarian cancer
Source: Cell Death Dis. 2022 Mar 24;13(3):263. doi: 10.1038/s41419-022-04670-7 (PMC8948190; doi:10.1038/s41419-022-04670-7)
Supplement: Supplementary file 3 — Highlights [file 41419_2022_4670_MOESM3_ESM.docx]

**Highlights:**

HMGB3 was highly expressed and indicated drug resistance in HGSOC.

HMGB3 facilitated the PARPi resistance of ovarian cancer *in vitro* and *in vivo*.

HMGB3 interacted with PARP1 in a PARylation-dependent manner.

HMGB3 promoted the PARylation activity of PARP1 by preventing PARP1 trapping.
