# Supplementary material for: HMGB3 promotes PARP inhibitor resistance through interacting with PARP1 in ovarian cancer
Source: Cell Death Dis. 2022 Mar 24;13(3):263. doi: 10.1038/s41419-022-04670-7 (PMC8948190; doi:10.1038/s41419-022-04670-7)
Supplement: Supplementary file 5 — Author contribution form [file 41419_2022_4670_MOESM5_ESM.pdf]

DECLARATION OF CONTRIBUTIONS TO ARTICLE

ADMC

Manuscript Number: CDDIS-21-3238

Journal Name: *Cell Death & Disease* (the 'Journal')

Proposed Title of the Contribution:

HMGB3 promotes PARP inhibitor resistance through interacting with PARP1 in ovarian cancer (the 'Contribution)

Author(s):

Hanlin Ma, Gonghua Qi, Fang Han, Wei Lu, Jiali Peng, Rongrong Li, Shi Yan, Cunzhong Yuan, Beihua Kong (the 'Authors')

For all *CDDis* articles, each person named as an author in the published version must be able to show he or she has contributed substantially to the article.

Authorship credit should be based on 1) substantial contributions to conception and design, acquisition of data, or analysis and interpretation of data; 2) drafting the article or revising it critically for important intellectual content; and 3) final approval of the version to be published. Authors should meet conditions 1, 2 and 3.

Any person who cannot be shown to have made a substantial contribution to the article cannot be listed as an author in the final version. The name of any person who is deemed to have made a minor contribution can, however, appear in the Acknowledgments section of the article.

Please complete the table below to indicate the contributions of all named authors to the manuscript.

Author Full Name:	Specification of Contribution to the Manuscript:
Hanlin Ma	Acquisition, analysis, and interpretation of data; Drafting and final approval of the article
Gonghua Qi	Analysis and interpretation of data; Technical support; Revision and final approval of the manuscript
Fang Han	Technical support; Revision and final approval of the manuscript
Wei Lu	Technical support; Revision and final approval of the manuscript
Jiali Peng	Interpretation of data; Technical support; Revision and final approval of the manuscript
Rongrong Li	Analysis and interpretation of data; Revision and final approval of the manuscript
Shi Yan	Analysis and interpretation of data; Revision and final approval of the manuscript
Cunzhong Yuan	Revision and final approval of the manuscript
Beihua Kong	Conception and design; Revision of the manuscript; Administrative, and material support; Final approval and Study supervision

Please complete the table below to indicate the contributions of all named authors to the figures.

Figure 1:

H.M generated the data and prepared panel A-E; G.Q generated the data and prepared panel F; J.P labelled the image; H.M assembled the figure.

Figure 2:

H.M generated the data; R.L labelled the imag; G.Q assembled the figure.

Figure 3:

H.M generated the data and assembled the figure; G.Q labelled the image.

Figure 4:

H.M generated the data; J.P labelled the image. G.Q assembled the figure.

Figure 5:

H.M generated the data and assembled the figure; G.Q labelled the image.

Figure 6:

H.M generated the data; S.Y and C.Y labelled the image. G.Q assembled the figure.

Signed for and on behalf of the Author(s):	Print Name:	Date:
Kong BeiHua	Beihua Kong	2021/12/21
